# Supplementary material for: Qualitative interview study of parents’ perspectives, concerns and experiences of the management of lower respiratory tract infections in children in primary care
Source: BMJ Open. 2017 Sep 15;7(9):e015701. doi: 10.1136/bmjopen-2016-015701 (PMC5640115; doi:10.1136/bmjopen-2016-015701)
Supplement: Supplementary file 1 [file bmjopen-2016-015701supp001.pdf]

Appendix 1: Interview guide

Section 1: presenting illness

- Before visiting the GP can you describe what happened/when you first noticed [name] was poorly?
- By the time you took [name] to the GP what particular symptoms did [name] have?
    - What, timings, together/separate?
- How did you feel about [name] being ill at the time?
- What was it that made you take [name] to the GP?
    - What was most concerning about the illness?
        - Eg compared to other coughs/illness
    - Had they had any previously similar episodes?
        - Generally quite well?
- Can you describe what you thought might be wrong with [name] at the time?
    - Read anything on the internet
    - Advice from friends/family/pharmacist
- How were you feeling in yourself?
    - Did [name’s] illness impact on the family?
    - Concerns re cross-infection?
- Before visiting the GP had you tried any form of treatment?
    - E.g Calpol, Nurofen?
    - Talk to other parents?
    - Generally do you wait and see/get medicines?
- What happened at your appointment?
    - What did the GP do?
    - Did they give you any medicines/advice (and if so what) /examine [name]?
- What did you hope to get from the GP appointment?
    - Reassure, prescribe antibiotics
- Can you think back and describe whether your hopes/expectations about the GP consultation were met?
    - Why / why not?
- If [name] had the same symptoms again how would you manage the situation next time?
    - Would you do anything differently?
        - Visit GP sooner?
        - Delay?

        - Not attend?
        - Make wishes more explicit with GP?
        - Reconsider value of Abs?

Section 2: understanding of antibiotics

- [Name] was/wasn't prescribed a/bs, how did you feel about that?
    - Why did you think a/bs were/were not needed?
    - E.g. Did you want [name] to have antibiotics?
    - How did antibiotics help [name] and your family get better?
        - Quicker recovery
        - Feel empowered
        - Could go back to school/playgroup?
    - How would you have felt if [name] had not received antibiotics?
        - Can you think of a time you have taken [name] to the GP and they weren't given a/bs?
        - Do you think the a/bs made [name] better?
        - Would they have got better themselves with no a/bs?
- Can you tell me about your thoughts about antibiotics and their use in children with chest infections?
    - E.g. effective/not-effective? Best avoided?
    - E.g. any concerns about giving antibiotics to children?
    - Side effects
    - Read anything of the Internet etc?
    - Concerns about sugar content/flavourings/colouring?
    - Taste issues?
- Have you heard of antibiotic resistance?
    - What does it mean to you?
    - Check understanding re resistant bacteria vs resistant body
- Have you heard about 'super-bugs'? Do you see this to be linked to prescribing antibiotics?
- Was it explained to you that you must finish all the antibiotics prescribed? And why it is important?

- [Remind parents that it does not matter if they are unsure, and provide information leaflet explaining antibiotic resistance after the interview.]

Recruitment/participation:

- A future trial is currently being developed to test how effective antibiotics are because we need to know which antibiotics work best in which children. We know that whilst most children get antibiotics, many children will probably settle without antibiotics but some will benefit from antibiotics. What we don't know is if antibiotics help or not and which children get benefit from antibiotics and which children suffer side effects (rashes, diarrhoea, resistance).
- The team are going to be doing a trial which would involve some children taking the medicine and others taking a dummy/sugar-coated pill, this would be randomly allocated. All the children will be fully monitored during the trial and will be carefully reviewed if they are not improving.
    - If you had the opportunity would you be interested in participating in this kind of trial?
    - What do you think about the possibility of your child taking the dummy pill?
- How did you feel about being contacted by letter?
    - Preferred GP/face to face?
