# Supplementary material for: Qualitative interview study of parents’ perspectives, concerns and experiences of the management of lower respiratory tract infections in children in primary care
Source: BMJ Open. 2017 Sep 15;7(9):e015701. doi: 10.1136/bmjopen-2016-015701 (PMC5640115; doi:10.1136/bmjopen-2016-015701)
Supplement: Supplementary file 2 [file bmjopen-2016-015701supp002.pdf]

Appendix 2: Exemplary Quotations

Themes	Illustrative quotations from participants
1. *Parental perspectives on infection*	
1.1 Parental concerns	1.1.1 “Oh, terrified. Because of the breathing, I was really frightened about – because the coughing – when she’s having these coughing fits – it’s just really scary and I was – I was scared to put her to bed because of the wheezing” (P4, 106-108)<br>1.1.2 “all you want to do is just stop the cough and you can’t” (P21, 83)<br>1.1.3 “Fine, obviously just a little bit concerned, that was all; but apart from that, I was fine – as long as she was getting the treatment that she needed, then I was happy” (P1, 71-72)<br>1.1.4 “she’d been unwell really for the biggest part of the beginning of the week” (P22, 19)<br>1.1.5 “I think my main concern was the fact that he wasn’t his usual – playful self” (P9, 34)<br>1.1.6 “where it’s – such a small body and it’s so hard for them to actually fight back” (P10, 142-3)<br>1.1.7 “It’s – the problem we have with [name] is it’s very hard to know when he is unwell because he just carries on and when – where he’s quite young as well – he won’t sit there and say, mummy my throat hurts, he’ll just carry on as normal” (P7, 30-32)<br>1.1.8. “You know, because he’s only little as well and at that age – because they can go downhill very quickly, that’s what I was worried about” (P8, 89-90).<br>1.1.9 “there was that fear that it could have – it could be developing something worse” (P13, 302-303)
1.2 Impact of child’s illness	1.2.1 “[son] did hear her coughing, so it did keep him awake as well” (P21, 313-314)<br>1.2.2 “she gets very out of breath very quickly, so she can’t join in her sports days, things like that; she doesn’t ever complete a full week at pre-school” (P2, 109-110)<br>1.2.3 “a lot of times parents are working, both parents, you need to get back to work; it costs money to have a child off sick and then you have to stay off sick, you lose a day’s pay or – or perhaps you have to pay a childminder, even though you’re not at work, you have to stay home from work as well” (P10, 286-289)
1.3 Symptoms	1.3.1 “He wasn’t his usual playful self, he was just sort of a bit off colour” (P9, 21-22)
1.4 Previously similar infections	1.4.1 “Well she’s had – antibiotics now for chest infections probably three or four times” (P14, 10)
2. *Parental perspectives on antibiotic use for lower respiratory tract infection*	
2.1 Benefits and efficacy	2.1.1 “If I didn’t give her the antibiotics, I think maybe she would of, but it probably would have tooken a lot, lot longer, but…no, I think the antibiotics helped. They helped – and they make it quicker as well, so they are not so ill for long” (P1, 173-175)<br>2.1.2 “anything you can do to help speed up the process and get her better, so that you can get back to normal, because it’s – it has such an impact” (P4, 327-329)<br>2.1.3 “I think the risks of something bad happening or, you know, in the night and not being able to breathe – would…yes, would have been more worrying and, you know maybe either I would have had to have called an ambulance or taken him into hospital or something like that. And I think that was definitely avoided by the antibiotics” (P5, 284-289)<br>2.1.4 “If there’s antibiotics that can fix whatever they’ve got then – yes – that they can sleep better and breath” (P5, 193-4)<br>2.1.5 “I don’t think there’s any point in letting a little baby cough like that and suffer like that for – I don’t know how long – I – worry that they won’t – not that they won’t get over eventually themselves, but – I think..that it’s my job to keep them well and – give them – yes – the opportunity to get better” (P5, 202-5)

	2.1.6 “They 100% work, 100%” (P1, 231)<br>2.1.7 “I mean they made [name] better in December, so, yes, they must be effective in children” (P3, 280-281)<br>2.1.8 “I do, yes. Antibiotics certainly helped both my children – but I think they are only effective if you use them correctly, so if you do finish the whole course of treatment” (P10, 275-276)<br>2.1.9 “should he be having antibiotics, you know – is it going to make any difference at all?” (P9, 85-86)
2.2 Parental concerns about problems with antibiotics	2.2.1 “no, upset like nasty nappies and things but – no – nothing, aside from that” (P18, 314-315).<br>2.2.2 “no, none that I’m aware of” (P23, 161)
3. *The GP appointment*	
3.1 Hopes and expectations	3.1.1 “Just to get her better really” (P21, 179)<br>3.1.2 “just that I wasn’t a neurotic mother, confirmation that there was something wrong with my child and something to fix her” (P17, 125-6).<br>3.1.3 “I was hoping for antibiotics. I was – I kind of knew that it was going to be antibiotics, again, just going from experience” (P7, 180-1).
3.2 Positive / negative experiences	3.2.1 “the lady we saw the next day was lovely; she really took the time to…you know, really – sort of – looked at everything and – seemed, you know, genuine, genuinely – sort of concerned for her and what have you” (P4, 214-217)<br>3.2.2 “I suffer with postnatal depression, so that’s why I like – she [GP] understands me as a mother and a person as well and there are a couple of other doctors that I like to see because they know the history” (P18, 173-176).
3.3 Access to health care	3.3.1 “It’s nice to know that I can – if I do have any questions, I can phone up and speak to the nurses and they’ve always said, you know, no question is too silly, we are always at the end of the phone” (P7, 305-7)
3.4 Advice	3.4.1 “Spoke to me through what to do, the best thing to do, you know, keeping him cool, giving me plenty of information as well” (P19, 190-191)<br>3.4.2 “I think she probably said that we’d see some improvement within about four or five days” (P14, 222-3)
4. *Decision making*	
4.1Agreement/disagreement for an antibiotic prescription	4.1.1 “I must admit there was a little bit of relief – because it meant I hadn’t overreacted and I think that she was poorly enough” (P3, 205-6).<br>4.1.2 “I think – I felt like she’d taken his illness seriously” (P10, 191).<br>4.1.3 “It felt like – good, you know something – you feel like something is being done to help the situation” (P4, 300-301).<br>4.1.4 “I know that he’d get better with them” (P20, 149)<br>4.1.5 “I was quite happy for her to have antibiotics to treat it because it was disturbing her sleep and showing no signs of dissipating by itself (P17, 35-36)<br>4.1.6 “…it was kind of expected really, neither happy nor unhappy” (P15, 153)<br>4.1.7 “But you don’t see them every day, every night, how the kid’s suffering or what they were doing, you know” (P12, 414-416)<br>4.1.8 “I was a bit shocked I thought, oh, right, okay, it’s a cough – because obviously there’s something not right, he’s got a cough” (P8, 229-230)<br>4.1.9 “I think it was upsetting because I was thinking that my child would be suffering” (P10, 218-9).<br>4.1.10 “I think I would have been a little frustrated because at that point I did know that it was – it was getting worse and I probably would have thought – well it’s going to need another trip to the doctor’s in a couple of days – which is just a waste of time for me and I probably would have just been quite annoyed” (P9, 239-242)<br>4.1.11 “they’d sent me away saying she just had a virus” (P4, 68-9)<br>4.1.12 “…I wasn’t convinced that they were right…really, because…if he’s going to clear it up on his own, it’s normally gone within three or four days […] But – yes, doctors know what they’re talking about, don’t they? You just go with it, don’t you – really” (P16, 234)<br>4.1.13 “So, like I said, I go by the doctor’s judgement to make it better for my daughter” (P1, 148-9)

	4.1.14 "I'd probably let them tell me what's best for her, really. They are the professionals, I'm not" (P21, 216-217)<br>4.1.15 "in some cases you don't feel like you can challenge a doctor because they think well, they are the doctor, they're trained and if I start kicking off and making a fuss, they're just going to get really annoyed with me and just think I'm an overprotective parent" (P7, 278-281).<br>4.1.16 "I was here [GP surgery] yesterday, they told me there is no chest infection: why do I have to wait for another night for him to be treated? If he had been given [antibiotics] yesterday, they could – he could have been better, you know" (P12, 322-325)
4.2 Perspectives of GP's prescribing behaviour	4.2.1 "I'm of the generation that – you know – if you've got an infection, go to the doctor and get some antibiotics. I know now that – that things are very different" (P22, 349-351)<br>4.2.2 "Um…is where your body can't fight – you like become immune to the antibiotics and it doesn't do anything?" (P13, 455-456)<br>4.2.3 "Yes, overprescribing antibiotics or prescribing them for things like coughs and colds – which they are ineffective against anyway…So that can lead to superbugs" (P10, 376-7).<br>4.2.4 "No, never heard of it" (P11, 176)<br>4.2.5 "I think, in my experience, there's a good balance here – about prescribing them when they are needed" (P5, 431-432)<br>4.2.6 "I have heard of people going to the GP for really minor things and getting antibiotics and I find that frustrating" (P10, 233-234).<br>4.2.7 "There was a thing on the news the other day, that they're…doctors are going to earn more money if they prescribe less antibiotics or something. That's wrong, isn't it?" (P16, 110-111).
4.3 Parental knowledge of LRTI and their management	4.3.1 "you know instantly if your child is not right" (P3, 72)<br>4.3.2 "I know that they are going to – tell you to wait a week, so I wait a week before I go in" (P16, 255-6).<br>4.3.3 "three days is too much continuously, then I have to bring them in" (P12, 399-400)<br>4.3.4 "when they are getting worse and no sign of getting better, then I would, without doubt, go" (P5, 242-243)<br>4.3.5 "I feel so guilty to think – oh God – I feel like I'm wasting their time" (P8, 223)<br>4.3.6 "You sometimes feel a little bit like…a neurotic mother" (P4, 211)<br>4.3.7 "Yes, I know the signs: he has them so often that I can sort of pick the signs and as soon as that sign appears" (P11, 38-9)<br>4.3.8 "As a parent, you think the worst; you start Googling. I think – we knew it was some kind of chest infection" (P13, 152-4).<br>4.3.9 "I'm a nurse, so I kind of have quite a good understanding of what was going on" (P9, 71).<br>4.3.10 "I did think she had a cold and obviously until the said what it is, then I was obviously a bit shocked" (P1, 68-9)<br>4.3.11 "antibiotics shouldn't just be given out for coughs and colds and viruses and things…I know that GPs often say – antibiotics are no good for viruses, it's only bacterial infections" (P10, 246-7)<br>4.3.12 "because obviously a chest infection is obviously a viral infection, isn't it, so I think most infections to need something to help" (P1, 132-133)
