# Supplementary material for: Qualitative interview study of parents’ perspectives, concerns and experiences of the management of lower respiratory tract infections in children in primary care
Source: BMJ Open. 2017 Sep 15;7(9):e015701. doi: 10.1136/bmjopen-2016-015701 (PMC5640115; doi:10.1136/bmjopen-2016-015701)
Supplement: Author's manuscript [file bmjopen-2016-015701.draft_revisions.pdf]

BMJ Open is committed to open peer review. As part of this commitment we make the peer review history of every article we publish publicly available.

When an article is published we post the peer reviewers' comments and the authors' responses online. We also post the versions of the paper that were used during peer review. These are the versions that the peer review comments apply to.

The versions of the paper that follow are the versions that were submitted during the peer review process. They are not the versions of record or the final published versions. They should not be cited or distributed as the published version of this manuscript.

BMJ Open is an open access journal and the full, final, typeset and author-corrected version of record of the manuscript is available on our site with no access controls, subscription charges or pay-per-view fees (http://bmjopen.bmj.com).

If you have any questions on BMJ Open's open peer review process please email editorial.bmjopen@bmj.com

BMJ Open

A qualitative interview study of parents' perspectives, concerns and experiences of the management of lower respiratory tract infections in children in primary care.

Journal:	*BMJ Open*
Manuscript ID	bmjopen-2016-015701
Article Type:	Research
Date Submitted by the Author:	04-Jan-2017
Complete List of Authors:	Halls, Amy; University of Southampton, Primary Care and Population Sciences<br>van't Hoff, Catherine; Royal Bournemouth and Christchurch Hospitals NHS Foundation Trust<br>Little, Paul; University of Southampton, Primary Care and Population Science;<br>Verheij, Theo; University Medical Center Utrecht, Julius Center for Health Sciences and Primary Care<br>Leydon, Gerry; University of Southampton, Primary Care and Population Sciences
**Primary Subject Heading**:	General practice / Family practice
Secondary Subject Heading:	Qualitative research
Keywords:	PRIMARY CARE, QUALITATIVE RESEARCH, Antibiotics, Interviews

SCHOLARONE™
Manuscripts

Title: A qualitative interview study of parents' perspectives, concerns and experiences of the management of lower respiratory tract infections in children in primary care.

Halls, AV* * van't Hoff, CI** * Little, P* Verhejj, TJM.[+] Leydon, GM.*

*University of Southampton, Faculty of Medicine, Academic Unit of Primary Care and Population Sciences, Aldermoor Health Centre, Aldermoor Close, Southampton, S016 5ST, United Kingdom.

**Education Centre, Royal Bournemouth Hospital, Castle Lane East, Bournemouth, BH7 7DW, United Kingdom.

[+]Department of General Practice, UMC Utrecht, div. Julius Centrum, Huispost Str. 6.131, PO BOS 85500, 3508 GA Utrecht, The Netherlands

*The first two authors should be regarded as joint First Authors.

Corresponding author:

Dr Gerry Leydon,

University of Southampton, Faculty of Medicine, Academic Unit of Primary Care and Population Sciences, Aldermoor Health Centre, Aldermoor Close, Southampton, S016 5ST, United Kingdom.

gerry@soton.ac.uk

Word count: 3905

For peer review only - http://bmjopen.bmj.com/site/about/guidelines.xhtml

For peer review only

Abstract

Objective: To explore parents' perspectives, concerns and experiences of the management of lower respiratory tract infections (LRTIs) in children in primary care.

Design: Qualitative semi-structured interview study.

Setting: UK primary care

Participants: 23 parents of children aged from six months to ten years presenting with LRTI in primary care.

Method: Thematic analysis of semi-structured interviews (either in person or by telephone) conducted with parents to explore their experiences and views on their children being prescribed antibiotics for LRTI.

Results: Four major themes were identified and these are: 1) perspectives on infection, 2) antibiotic use, 3) the GP appointment, and 4) decision-making around prescribing. Symptomatic relief was a key concern: most troublesome symptoms were cough and perceived breathing difficulty, but also fever and malaise. Many parents were reluctant to use self-care medication, tended to support antibiotic use, and signalled a belief they are effective for symptoms, illness duration and for preventing complications. However, parental expectations varied from a desire for reassurance and advice to an explicit preference for an antibiotic prescription. These preferences were shaped by: 1) the age of the child, with younger children perceived as more vulnerable because of their greater difficulty in communicating, and concerns about rapid deterioration; 2) the perceived severity of the illness; and 3) disruption to daily routine. When there was disagreement with the GP parents described feeling dismissed, and they were critical of inconsistent prescribing when they re-consult. When agreement between the parent and doctor featured, parents described a feeling of relief and legitimation for consulting, feeling reassured that the illness did indeed warrant a doctor's attention.

Conclusion: Symptomatic relief is a major concern for parents. Careful exploration of expectations, and eliciting worries about key symptoms and impact on daily life will be needed to help parents understand a no (or delayed) antibiotic recommendation.

For peer review only

Strengths and limitations of this study

- Provides a detailed view of key experiences and understandings of a sample of parents of children presenting with symptoms of LRTI in southern England.
- Only two fathers included (compared with 23 mothers) so the views of fathers were not fully captured.
- Semi-structured interviews were the optimal data collection method given the aims of the research and analytical saturation was reached.

For peer review only

For peer review only - http://bmjopen.bmj.com/site/about/guidelines.xhtml

BACKGROUND

The majority of respiratory tract infections (RTIs) are viral[1, 2] and will resolve spontaneously with analgesia and rest. Infections and treatment with antibiotics are important health concerns[3]. A third of children presenting to a GP with febrile illness receive an antibiotic prescription[4]. NICE (National Institute for Health and Care Excellence) guidance recommends patients be given no antibiotic prescription or a delayed prescription (to be fulfilled if symptoms worsen)[5], as well as advice about expected time course of infection and how to manage symptoms. Patients' education and general understanding of the medications they are prescribed affect compliance[6] and with antibiotics this could lead to the development of antibiotic resistant bacteria[6, 7]. A study of children under 4 suggested that parents often believed antibiotics were indicated for cough and upper respiratory tract infections[8], yet most parents were aware of antibiotic resistance and its complications[8, 9] or expressed concern about the overuse of antibiotics. However, it has been suggested more than half of adults expect an antibiotic prescription when attending with symptoms of RTI.[6]

Uncomplicated acute lower respiratory tract infection is the commonest acute infection[10], and is likely to have the longest and highest symptom burden of all acute RTIs[11]. Previous work has assessed the key concerns about symptoms of adults presenting with cough, but did not address the concerns about symptoms of parents with children[12].

We report a qualitative study of the key concerns of parents about symptoms of their children presenting with acute LRTI in primary care and their perceptions about antibiotics and the consultation.

METHODS

Participants and procedure

Practices were identified through the Primary Care Research Network and a poster presentation at the Wessex Research Sites Initiative Conference. Practices searched their databases for children aged 6 months to 12 years who had presented to primary care with symptoms of LRTI between January 2013 and March 2015. Parents were interviewed regardless of whether antibiotics were prescribed.

For peer review only - http://bmjopen.bmj.com/site/about/guidelines.xhtml

Interviews

All parents who agreed to participate were interviewed. Two interviewers (CvH and AH) conducted face to face (n=20) or telephone interviews (n=3), each lasting approximately 30 to 60 minutes, with an average duration of 42 minutes. All interviews were audio-recorded and transcribed verbatim. Qualitative interviews provided the best method for gathering insights into parents' views about their children's experiences of RTI and its management in primary care. The semi-structured interview guide (Appendix 1) included key topic areas whilst also giving flexibility to explore new areas if they arose.

Analysis

Inductive thematic analysis[(13)] was conducted on all transcripts to gain an understanding of the perspectives, attitudes and concerns of parents regarding the managing of LRTI in children in primary care. Familiarisation was achieved following immersion in the transcripts. Consistently occurring patterns and themes in the data were identified and labelled with codes: these code labels referred to the operationalisation of the theme content. A label and full description were provided for each theme. These codes and definitions were refined during a continuous process: this involved themes being linked, grouped, moved, re-labelled, added and removed as appropriate in order to produce a set of themes, subthemes, and a coding manual. The coding manual thoroughly explained all the data. The coding was iteratively developed, led by GL, CvH and AH.

Participants In total 23 participants took part in this study. In all interviews the mother of the child was interviewed, and fathers were present in two interviews. The age of the mothers[1] ranged from 21 to 47 (median age 34) and the age of the children ranged from 6 months to 10 years (median age 1 year 9 months) (see Table 1 for participant characteristics).

[1] The age of the fathers was not recorded.

For peer review only - http://bmjopen.bmj.com/site/about/guidelines.xhtml

Table 1: Participant characteristics

FINDINGS

Thematic analysis identified four themes relating to factors which enhanced our understanding of parents' perspectives, concerns and experiences of the management of LRTI in children in primary care. Table 2 outlines the themes and subthemes and these are used to structure the findings.

Table 2: Themes identified in analysis

Themes	Sub-themes
1. Parental perspectives on infection	1.1 Symptoms<br>1.2 Previously similar infections<br>1.3 Parental concerns<br>1.4 Impact of child's illness<br>1.5 Home management strategies and medicine usage
2. Parental perspectives on antibiotic use for lower respiratory tract infection	2.1 Benefits and efficacy<br>2.2 Parental concerns
3. The GP appointment	3.1 Hopes and expectations<br>3.2 Positive/negative experiences<br>3.3 Access to health care<br>3.4 Advice
4. Decision making	4.1 Agreement/disagreement for an AB prescription<br>4.2 Perspectives of GP's prescribing behaviour<br>4.3 Parental knowledge of LRTI and their management

The following sections describe each theme in turn. Numbers in parentheses refer to the theme, subtheme, and relevant exemplary quotation provided in Appendix 2.

For peer review only - http://bmjopen.bmj.com/site/about/guidelines.xhtml

For peer review only

1. Parental perspectives on infection

Parental concerns: Parents spoke in detail about their concerns when their child was ill. By far the strongest and most frequent concern centred on breathing difficulties and airway limitations (1.1.1). Other concerns included general concerns or unhappiness that their child was unwell (1.1.2). However, some mothers were less concerned and this was associated with factors such as the child not being their first child, or if the child was perceived to be receiving appropriate treatment (1.1.3). Parents reported worry about symptoms their child had, including the duration of symptoms (1.3.4) or the qualitative difference in the child's behaviour (1.1.5).

Parents of younger children (two years and under) generally had more concerns due to their child's young age. These concerns centred on three areas: increased vulnerability (1.1.6); difficulties in communicating (1.1.7); and the potential for rapid deterioration (1.1.8). Some parents also expressed worry about complications (1.1.9).

Impact of child's illness: Parents were directly asked about the impact of the child's illness on them and their family and readily gave lack of sleep and tiredness across the family as the primary impact (1.2.1). Parents of children with underlying health conditions spoke in more detail about the impact of their child's condition (1.2.2). Working mothers spoke of social implications such as needing additional time off work and the subsequent financial impact (1.2.3). These pressures were also reflected when mothers talked about the need for antibiotics to shorten illness duration (see section 2.1).

Symptoms: All symptoms reported by parents are summarised in Table 3, which shows that the most commonly reported symptom by the study participants was a cough, but breathing, fever and malaise were also very commonly reported as troublesome symptoms.

Table 3: Reported symptoms

Symptoms	Frequency
Cough	18
Breathing difficulties	16
Fever/temperature	16
Malaise*	15
Difficulty sleeping	11

For peer review only - http://bmjopen.bmj.com/site/about/guidelines.xhtml

Feeding problems/appetite	10
Wheeze	10
Runny nose	9
Fatigue/tiredness	6
Vomit	6
Sore throat/hoarse voice	5
Ear ache	2
Pain	2
Headache	1
Fainting	1
Heart racing	1
Rash	1

*Malaise was used as a term to categorise anything parents said referring to a change in behaviours, character, or appearing unwell (1.1.1).

Previously similar infections: During the course of the interview parents recounted previous infections or the prolonged nature of the infection in question (1.2.1). Mothers of children with underlying health conditions often spoke of this in detail, but it is not central to the current study and so is not reported.

Home management strategies and medicine usage: Parents initially dealt with their child's infection in different ways. Parents reported trying home management strategies (see Table 5) or complementary medicines before visiting the GP (1.5.1). Parents' views on over-the-counter medicines were varied (1.5.2) although many parents used Calpol and Nurofen to relieve symptoms, most commonly a temperature. Perhaps unsurprisingly many parents reported reluctance to use medications unless they feel it is strongly needed or recommended by a doctor (1.5.3).

Parents initially dealt with their child's infection in different ways. Parents reported trying home management strategies or complementary medicines before visiting the GP (see Table 4).

Table 4: Home management strategies tried in addition to Calpol / Nurofen:

**Strategy**	**Interview mentioned in**
Steam inhalation	4,5,6,8,10,11,13,16
Vaporisers/scents	6,8,13,16

For peer review only - http://bmjopen.bmj.com/site/about/guidelines.xhtml

Throat pastilles/cough syrup	3,4,12,14,16,17
Drinks	3,4,8,12,19,21
Cold strategies	13,14,16
Raise the bed	8,16,17,19,23
Vicks	5,8,10,16, 21
Improved diet	5
Other*	2
No additional home management	1,7,9,15,20,22

*Participant 2's child took daily prophylactic antibiotics for a severe undiagnosed health condition: their home management strategy was to give a double dose, as recommended by the specialist.

2. Parental perspectives on antibiotic use for lower respiratory tract infection

Parents spoke in detail about their perceptions of antibiotic use, their attitudes to prescribing and their concerns about antibiotics. Their views were varied but overall parents were happy with their experiences of antibiotic usage in their children.

Benefits and efficacy: Parents cited antibiotics as having three main benefits. Firstly, shortening illness duration (2.1.1), which had the additional benefit of helping parents to return to work (2.1.2). Secondly, antibiotics were described as being able to prevent complications (2.1.3). Thirdly, antibiotics were thought to provide symptomatic relief (2.1.4-2.1.5). Most parents expressed certainty that antibiotics were effective (2.1.6) and they often used their experience of their child recovering during past episodes to infer the effectiveness of antibiotics (2.1.7), although one parent did appreciate there were conditions in which antibiotics are more effective (2.1.8). In contrast, a minority of parents were aware that antibiotics are not always effective while some volunteered awareness of the lack of evidence for prescribing in children (2.1.9).

Parental concerns: Parental concerns about antibiotic use were predominantly about the composition of antibiotic medication (taste, texture, smell, and use of additives). Many parents commented negatively on the taste and texture whereas views on additives such as colourings were more varied. Generally parents had few concerns about adverse effects (2.2.1), especially when their child had not experienced any following previous use (2.2.2).

3. The GP appointment

Hopes and expectations: Parent interviews indicated varied hopes and expectations of the outcome of their GP appointments. Many parents described a desire for symptomatic relief,

For peer review only - http://bmjopen.bmj.com/site/about/guidelines.xhtml

reassurance and / or advice (3.1.1, 3.1.2) while some, importantly in the minority, attended the GP consultation with an explicit wish for an antibiotic prescription (3.1.3).

Positive and negative experiences:

Many parents had positive views of the GP appointment; in particular when GPs were perceived to take time for a thorough assessment of the child (3.2.1) or with GPs who have built a history with the family (3.2.2). A physical examination has previously been shown to be a key expectation of parents[9]. However, some parents used the interviews as an opportunity to voice frustrations over 'bad' appointments; these centred around feeling rushed, appointments running late or parental concerns not being taken seriously. Differences in parental and GP opinions also contributed to dissatisfaction (discussed further under theme 4).

Access to health care: Parents reported irritation about difficulties in getting appointments. They also discussed a lack of continuity of care, which could mean that deterioration was not noticed by the GP and parents had to recount their story on multiple occasions. Many spoke of needing to use out of hours services, and this brought with it new concerns around queueing, and seeing an unfamiliar doctor in an unfamiliar environment. One parent, living rurally, spoke of concerns around rapid deterioration out of normal working hours. However, many parents spoke positively of the use of the NHS 111 service and their advice. Parents of children with underlying health conditions spoke of easy access to medical expertise (3.3.1).

Advice: Parents were asked to discuss advice they were given at the index appointment. They primarily volunteered advice on home-management approaches they had already tried prior to the GP consultation (3.4.1). Many parents did not recall being given specific advice about the natural history of the illness. Of those interviewed some were asked to re-consult if there was no improvement and some recalled receiving advice on how long symptoms would last (3.4.2).

4. Decision making

A shared decision making process between parent and GP, or an explanation of why antibiotics were not necessary, usually led to a more acceptable consultation when parents did not explicitly express a desire for antibiotics.

For peer review only - http://bmjopen.bmj.com/site/about/guidelines.xhtml

GP-parents agreement and disagreement about the need for an antibiotic prescription: When there was agreement about the need for a prescription between GP and parents, many parents described a feeling of relief. A prescription seemed to give parents the feeling of illness legitimation[1](4.1.1, 4.1.2). In addition to this, parents reported feeling empowered by a prescription because it signalled a positive action taken to help their child (4.1.3). Parents also described that they were pleased because their child would now have symptomatic relief (4.1.4, 4.1.5). However, parents of children with frequent chest infections or underlying health conditions did not seem to have strong opinions, viewing an antibiotic prescription as a necessary requirement (4.1.6).

When parents felt there was disagreement between them and their doctor, feelings of frustration were described. Frustration that doctors did not agree with their perception of the severity of infection (4.1.7) or that doctors did not think antibiotics were indicated (4.1.8). Parents felt frustrated and upset at the lack of symptomatic relief or that they would need to re-consult (4.1.9, 4.1.10).

Parents spoke about feeling dismissed by their GP, especially if the infection was thought to be viral (4.1.11). Parents reported uncertainty when they disagreed with the doctor's decision, but this narrative was balanced by belief in ultimately trusting the doctor's judgement and their professional expertise (4.1.12-4.1.14).

Some parents described discomfort and the feeling they could not question the GP's judgement (4.1.15). The majority of parents trusted doctors' expertise but many parents recounted occasions when they had not been prescribed antibiotics initially and then had re-consulted shortly after and been given a prescription. By having to re-consult, parents felt doctors were not consistent, and that their persistence or pestering would result in a prescription. This seemed to be often closely related to dissatisfaction with the GP appointment (4.1.16).

Perspective of GP's prescribing behaviour: Many parents had an awareness of overprescribing antibiotics and its consequences and some commented on doctors clearly trying to limit prescribing (4.2.1). Many parents understood antibiotic resistance to be an idea that over-use would result in your body becoming immune to the antibiotic; an idea which is echoed in the existing literature (4.2.2).

For peer review only - http://bmjopen.bmj.com/site/about/guidelines.xhtml

Parental concerns about over-use were reflected in their understanding of antibiotic resistance and superbugs. For example, parents with more accurate scientific knowledge often had a clear appreciation of the consequences of resistance (4.2.3). However, some parents were quite clear that they had very limited understanding of the concept of antibiotic resistance (4.2.4).

Parents offered both positive and negative opinions on GP's prescribing behaviours (4.2.5). Negative opinions of prescribing behaviour were often from word of mouth. For example, a few parents spoke about factors they believed to influence prescribing (4.2.6) such as believing doctors earn more if they prescribe fewer antibiotics (4.2.7).

Parental understanding of LRTI and their management: Parents had tacit knowledge of a suggested diagnosis, when to present, and if they felt antibiotics were indicated. Many parents spoke of knowing their child best (4.3.1), although this contrasted with previous comments, where parents described difficulties in communicating with young children. Parents appeared to have had different 'tipping points' with regard to help-seeking, such as when to present. Some parents tended to have a particular amount of time they were happy waiting before they consulted to help ensure they received antibiotics (i.e. illness duration would be such that a GP would be more likely to think antibiotics are required) (4.3.2). Others waited a specific number of days (4.3.3) or used a lack of improvement as a reason to consult (4.3.4). Although many parents had clear triggers for consulting, they simultaneously reported concerns about wasting doctors' time (4.3.5), or appearing over-anxious (4.3.6).

Parents often described having a good idea of the diagnosis before presenting. Parental knowledge of a diagnosis came from a variety of experiences: knowing their child, and prior experience, was a chief way of gauging severity and the need to consult (4.3.7). Other parents had conducted internet research to find a diagnosis (4.3.8). Others relied on their professional expertise (4.3.9) whereas some parents described not having an idea of the diagnosis (4.3.10).

There was some confusion over the role of antibiotics in treating LRTI. Some parents understood that antibiotics were only indicated for bacterial infections (4.3.11), whilst others

For peer review only

For peer review only - http://bmjopen.bmj.com/site/about/guidelines.xhtml

were unclear of the difference between bacterial and viral infections and did not know when antibiotics were indicated (4.3.12).

DISCUSSION

The study identified four themes that related to parents' perspectives, attitudes and concerns regarding the management of LTRI in children in primary care.

Main findings

Overall, this study provides insight into the views of parents of children who have consulted with their GP for suspected LRTI. It presents up to date evidence of the most significant concerns for parents, primarily sudden deterioration, breathing difficulties or the child's young age. Whilst most believed that antibiotics were effective for the symptoms of LRTI, many parents did not present with a certain expectation for antibiotics, and yet many felt frustration if the GP did not agree with their perception of the need to actively treat their child. There was a tension between parents wanting symptomatic relief for their individual child, the doctor's expertise and their disposal to not prescribe based on their understanding of need in the population in general.

When asked, many parents did not recall being given specific advice on the infection duration. However, interviews revealed that parents did have some understanding about likely duration of a LRTI as shown through the 'tipping points' discussed previously. NICE guidance[(5)] advises patients should be advised about the natural history of RTIs including usual total length. Interviews suggest this is important for GPs to emphasise so parents can be reassured about when to present.

Comparison with existing literature

Clinicians prescribe for medical indication and other reasons such as perceived pressure from parents[(1)] but this pressure is not always evident. Inappropriate antibiotic prescriptions may result in problems such as antimicrobial resistance[(3)] as well as the medicalisation of a self-limiting illness[(14)]. Whilst this study showed many parents wanted antibiotics, some indicated that they were happy to withhold antibiotic treatment and continue with non-medical self-management strategies and over the counter medicines in a context of reassurance, and when given a sense of illness legitimation. Parental satisfaction was likely to be highest when the GP was perceived to be thorough, that they were listened to and had their concerns taken

For peer review only - http://bmjopen.bmj.com/site/about/guidelines.xhtml

seriously. Where appropriate, having the medical history of the child or family taken into account also helped with parental satisfaction.

This study shows that parents often waited and consulted when they had reached a ‘tipping point’ at which they felt the severity and duration of the child’s illness was such that some action in the form of prescribing antibiotics was warranted. This resonates with research into urinary tract infections[15] in which respondents described an initial reluctance to consult but a visit was eventually prompted by symptom severity, often in conjunction with duration. Previous research has identified a need to knowledge share with patients to ensure clear understanding of antibiotics and their appropriate use[16]. However, this study also showed that even if parents had a good understanding of LRTI and its causes, the perceived vulnerability of their child could influence them to present sooner than they may have done had the child been older and more able to communicate how they were feeling.

For peer review only

IMPLICATIONS FOR CLINICAL PRACTICE

Overall, most parents were satisfied with their child’s GP consultation. Higher satisfaction was evident in parent narratives when parents believed their concerns about their child’s health had been taken seriously, from a thorough examination to having more time spent with them. Increased GP sensitivity to the wider concerns of parents and the need to employ communication strategies that encourage parental participation and validation of their concerns could ease the path towards improved parent/patient and GP antibiotics discussions and ultimately towards more prudent antibiotic prescribing and use.

The interviews showed that whilst some parents did present to their GP with the expectation of receiving an antibiotic prescription, many parents did not: other aims for the consultation included illness legitimisation and advice for symptomatic relief using home management strategies. GPs should know that parents are often willing to accept a treatment other than antibiotics, and indeed would rather have an alternative. This often correlated with the knowledge parents had about the causes of LRTI and appropriate treatment. Across the interviews, parents displayed varying levels of understanding and beliefs about causes, duration and treatment of LRTI and when they should present. Clarifying patient information, and helping to enhance general understanding, could help to reduce unnecessary GP consultations and smooth the interactional path when parents do consult.

For peer review only - http://bmjopen.bmj.com/site/about/guidelines.xhtml

STRENGTHS AND LIMITATIONS

The sample of participants is not representative of the wider population and just sensitises us to key experiences and understandings of a sample of parents. Participants may have had stronger opinions or other reasons for being interviewed than 'typical' or non-participant parents. Only two fathers were included in interviews (compared with 23 mothers) so fathers' views were not fully captured in this study. Interviews occurred at varied times in relation to the infection/index consultation for the consulting 'child', which means that some parents had differing recall demands. Finally, interviews provide insight into perspectives on events not a direct window to the event themselves (such as the GP appointment). However, semi-structured interviews were the optimal data collection method given the aims of this research project.

CONCLUSION

This study aimed to explore factors important to parents in the management of LTRI in children in primary care. Parental views were varied, but clearly indicated that symptomatic relief is a key consideration for their decision-making regarding when to consult and management preferences. Parents do not necessarily expect a prescription and a satisfactory consultation can be achieved by being perceived as thorough and in legitimating parents' reason for consulting. There is an on-going need for GPs to carefully explore concerns and expectations, and to tailor advice, information and reassurance for parents - particularly addressing the natural history, and the likely impact of antibiotics on symptom severity, duration and complications. Current research led by one of the authors (GL) is currently analysing video recorded UK GP consultations to understand the communication practices routinely engaged by patients and GPs when discussing the need or otherwise for antibiotic medication.

Acknowledgments

Interviewees for their invaluable contributions on this important topic.

NIHR Fellowship funding for Geraldine M Leydon during the conduct of this research.

Funding

For peer review only - http://bmjopen.bmj.com/site/about/guidelines.xhtml

This project was funded by the National Institute for Health Research Health Technology Assessment Programme (project number 13/34/64).

Department of Heath disclaimer:

The views and opinions expressed therein are those of the authors and do not necessarily reflect those of the Health Technology Assessment Programme, NIHR, NHS or the Department of Health.

No further data are available.

There are no competing interests.

Contributors:

GML was responsible for all aspects of the study including design, data analysis and interpretation, and manuscript preparation. AH and CvH collected, analysed and interpreted the data, and prepared the manuscript led by GL. PL is Principal Investigator of a larger affiliated study Antibiotics for lower Respiratory Tract Infection in Children presenting in Primary Care (ARTIC PC) and PL and TV were involved in the generation of the study idea and design, and manuscript comments and revisions.

References:

1. Peters S, Rowbotham S, Chisholm A, Wearden A, Moschogianis S, Cordingley L, et al. Managing self-limiting respiratory tract infections: a qualitative study of the usefulness of the delayed prescribing strategy. Br J Gen Pract. 2011;61(590):e579-89.
2. Thompson PL, Spyridis N, Sharland M, Gilbert RE, Saxena S, Long PF, et al. Changes in clinical indications for community antibiotic prescribing for children in the UK from 1996 to 2006: will the new NICE prescribing guidance on upper respiratory tract infections just be ignored? Arch Dis Child. 2009;94(5):337-40.
3. Davies S, Fowler T, Watson J, Livermore D, Walker D. Annual Report of the Chief Medical Officer: infection and the rise of antimicrobial resistance. Editorial Material. London: Department of Health; 2011 MAY 11 2013. Report No.: 0140-6736 Contract No.: 9878.
4. Moore M. Antibiotics: time to act. Br J Gen Pract. 2013;63(612):340-1.
5. National Institute for Health and Clinical Excellence: Guidance. Respiratory Tract Infections - Antibiotic Prescribing: Prescribing of Antibiotics for Self-Limiting Respiratory Tract Infections in Adults and Children in Primary Care. London: National Institute for Health and Clinical Excellence (UK)National Institute for Health and Clinical Excellence.; 2008.
6. McNulty CA, Nichols T, French DP, Joshi P, Butler CC. Expectations for consultations and antibiotics for respiratory tract infection in primary care: the RTI clinical iceberg. Br J Gen Pract. 2013;63(612):e429-36.

For peer review only - http://bmjopen.bmj.com/site/about/guidelines.xhtml

7. Little P, Stuart B, Moore M, Coenen S, Butler CC, Godycki-Cwirko M, et al. Amoxicillin for acute lower-respiratory-tract infection in primary care when pneumonia is not suspected: a 12-country, randomised, placebo-controlled trial. Lancet Infect Dis. 2013;13(2):123-9.
8. Trepka MJ, Belongia EA, Chyou PH, Davis JP, Schwartz B. The effect of a community intervention trial on parental knowledge and awareness of antibiotic resistance and appropriate antibiotic use in children. Pediatrics. 2001;107(1):E6.
9. Francis N, Phillips R, Wood F, Hood K, Simpson S, Butler C. Parents' and clinicians' views of an interactive booklet about respiratory tract infections in children: a qualitative process evaluation of the EQUIP randomised controlled trial. Bmc Family Practice. 2013;14.
10. Gulliford MC, Dregan A, Moore MV, Ashworth M, Staa T, McCann G, et al. Continued high rates of antibiotic prescribing to adults with respiratory tract infection: survey of 568 UK general practices. BMJ Open. 2014;4(10):e006245.
11. Thompson M, Vodicka TA, Blair PS, Buckley DI, Heneghan C, Hay AD, et al. Duration of symptoms of respiratory tract infections in children: systematic review. BMJ. 2013;347:f7027.
12. Cornford CS. Why patients consult when they cough: a comparison of consulting and non-consulting patients. Br J Gen Pract. 1998;48(436):1751-4.
13. Braun V, Clarke V. Using thematic analysis in psychology. Qualitative Research in Psychology [Internet]. 2006 05.01.15; 3(2):[77-101 pp.].
14. Andrews T, Thompson M, Buckley DI, Heneghan C, Deyo R, Redmond N, et al. Interventions to Influence Consulting and Antibiotic Use for Acute Respiratory Tract Infections in Children: A Systematic Review and Meta-Analysis. Plos One. 2012;7(1).
15. Leydon GM, Turner S, Smith H, Little P, team U. Women's views about management and cause of urinary tract infection: qualitative interview study. BMJ. 2010;340:c279.
16. Brookes-Howell L, Elwyn G, Hood K, Wood F, Cooper L, Goossens H, et al. 'The Body Gets Used to Them': Patients' Interpretations of Antibiotic Resistance and the Implications for Containment Strategies. Journal of General Internal Medicine. 2012;27(7):766-72.

For peer review only

For peer review only - http://bmjopen.bmj.com/site/about/guidelines.xhtml

Appendix 1: Interview guide

Section 1: presenting illness

- Before visiting the GP can you describe what happened/when you first noticed [name] was poorly?
- By the time you took [name] to the GP what particular symptoms did [name] have?
    - What, timings, together/separate?
- How did you feel about [name] being ill at the time?
- What was it that made you take [name] to the GP?
    - What was most concerning about the illness?
        - Eg compared to other coughs/illness
    - Had they had any previously similar episodes?
        - Generally quite well?
- Can you describe what you thought might be wrong with [name] at the time?
    - Read anything on the internet
    - Advice from friends/family/pharmacist
- How were you feeling in yourself?
    - Did [name's] illness impact on the family?
    - Concerns re cross-infection?
- Before visiting the GP had you tried any form of treatment?
    - E.g Calpol, Nurofen?
    - Talk to other parents?
    - Generally do you wait and see/get medicines?
- What happened at your appointment?
    - What did the GP do?
    - Did they give you any medicines/advice (and if so what) /examine [name]?
- What did you hope to get from the GP appointment?
    - Reassure, prescribe antibiotics
- Can you think back and describe whether your hopes/expectations about the GP consultation were met?
    - Why / why not?
- If [name] had the same symptoms again how would you manage the situation next time?
    - Would you do anything differently?
        - Visit GP sooner?
        - Delay?

For peer review only

- Not attend?
- Make wishes more explicit with GP?
- Reconsider value of Abs?

Section 2: understanding of antibiotics

- [Name] was/wasn't prescribed a/bs, how did you feel about that?
    - Why did you think a/bs were/were not needed?
    - E.g. Did you want [name] to have antibiotics?
    - How did antibiotics help [name] and your family get better?
        - Quicker recovery
        - Feel empowered
        - Could go back to school/playgroup?
    - How would you have felt if [name] had not received antibiotics?
        - Can you think of a time you have taken [name] to the GP and they weren't given a/bs?
        - Do you think the a/bs made [name] better?
        - Would they have got better themselves with no a/bs?
- Can you tell me about your thoughts about antibiotics and their use in children with chest infections?
    - E.g. effective/not-effective? Best avoided?
    - E.g. any concerns about giving antibiotics to children?
    - Side effects
    - Read anything of the Internet etc?
    - Concerns about sugar content/flavourings/colouring?
    - Taste issues?
- Have you heard of antibiotic resistance?
    - What does it mean to you?
    - Check understanding re resistant bacteria vs resistant body
- Have you heard about 'super-bugs'? Do you see this to be linked to prescribing antibiotics?
- Was it explained to you that you must finish all the antibiotics prescribed? And why it is important?

- [Remind parents that it does not matter if they are unsure, and provide information leaflet explaining antibiotic resistance after the interview.]

For peer review only - http://bmjopen.bmj.com/site/about/guidelines.xhtml

Appendix 2: Exemplary Quotations

Themes	Illustrative quotations from participants
1. Parental perspectives on infection	
1.1 Parental concerns	1.1.1 “Oh, terrified. Because of the breathing, I was really frightened about – because the coughing – when she’s having these coughing fits – it’s just really scary and I was – I was scared to put her to bed because of the wheezing” (P4, 106-108)<br>1.1.2 “all you want to do is just stop the cough and you can’t” (P21, 83)<br>1.1.3 “Fine, obviously just a little bit concerned, that was all; but apart from that, I was fine – as long as she was getting the treatment that she needed, then I was happy” (P1, 71-72)<br>1.1.4 “she’d been unwell really for the biggest part of the beginning of the week” (P22, 19)<br>1.1.5 “I think my main concern was the fact that he wasn’t his usual – playful self” (P9, 34)<br>1.1.6 “where it’s – such a small body and it’s so hard for them to actually fight back” (P10, 142-3)<br>1.1.7 “It’s – the problem we have with [name] is it’s very hard to know when he is unwell because he just carries on and when – where he’s quite young as well – he won’t sit there and say, mummy my throat hurts, he’ll just carry on as normal” (P7, 30-32)<br>1.1.8. “You know, because he’s only little as well and at that age – because they can go downhill very quickly, that’s what I was worried about” (P8, 89-90).<br>1.1.9 “there was that fear that it could have – it could be developing something worse” (P13, 302-303)
1.2 Impact of child’s illness	1.2.1 “[son] did hear her coughing, so it did keep him awake as well” (P21, 313-314)<br>1.2.2 “she gets very out of breath very quickly, so she can’t join in her sports days, things like that; she doesn’t ever complete a full week at pre-school” (P2, 109-110)<br>1.2.3 “a lot of times parents are working, both parents, you need to get back to work; it costs money to have a child off sick and then you have to stay off sick, you lose a day’s pay or – or perhaps you have to pay a childminder, even though you’re not at work, you have to stay home from work as well” (P10, 286-289)
1.3 Symptoms	1.3.1 “He wasn’t his usual playful self, he was just sort of a bit off colour” (P9, 21-22)
1.4 Previously similar infections	1.4.1 “Well she’s had – antibiotics now for chest infections probably three or four times” (P14, 10)
2. Parental perspectives on antibiotic use for lower respiratory tract infection	
2.1 Benefits and efficacy	2.1.1 “If I didn’t give her the antibiotics, I think maybe she would of, but it probably would have tooken a lot, lot longer, but…no, I think the antibiotics helped. They helped – and they make it quicker as well, so they are not so ill for long” (P1, 173-175)<br>2.1.2 “anything you can do to help speed up the process and get her better, so that you can get back to normal, because it’s – it has such an impact” (P4, 327-329)<br>2.1.3 “I think the risks of something bad happening or, you know, in the night and not being able to breathe – would…yes, would have been more worrying and, you know maybe either I would have had to have called an ambulance or taken him into hospital or something like that. And I think that was definitely avoided by the antibiotics” (P5, 284-289)<br>2.1.4 “If there’s antibiotics that can fix whatever they’ve got then – yes – that they can sleep better and breath” (P5, 193-4)<br>2.1.5 “I don’t think there’s any point in letting a little baby cough like that and suffer like that for – I don’t know how long – I – worry that they won’t – not that they won’t get over eventually themselves, but – I think..that it’s my job to

For peer review only - http://bmjopen.bmj.com/site/about/guidelines.xhtml

	keep them well and – give them – yes – the opportunity to get better" (P5, 202-5)<br>2.1.6 "They 100% work, 100%" (P1, 231)<br>2.1.7 "I mean they made [name] better in December, so, yes, they must be effective in children" (P3, 280-281)<br>2.1.8 "I do, yes. Antibiotics certainly helped both my children – but I think they are only effective if you use them correctly, so if you do finish the whole course of treatment" (P10, 275-276)<br>2.1.9 "should he be having antibiotics, you know – is it going to make any difference at all?" (P9, 85-86)
2.2 Parental concerns about problems with antibiotics	2.2.1 "no, upset like nasty nappies and things but – no – nothing, aside from that" (P18, 314-315).<br>2.2.2 "no, none that I'm aware of" (P23, 161)
3. *The GP appointment*	
3.1 Hopes and expectations	3.1.1 "Just to get her better really" (P21, 179)<br>3.1.2 "just that I wasn't a neurotic mother, confirmation that there was something wrong with my child and something to fix her" (P17, 125-6).<br>3.1.3 "I was hoping for antibiotics. I was – I kind of knew that it was going to be antibiotics, again, just going from experience" (P7, 180-1).
3.2 Positive / negative experiences	3.2.1 "the lady we saw the next day was lovely; she really took the time to…you know, really – sort of – looked at everything and – seemed, you know, genuine, genuinely – sort of concerned for her and what have you" (P4, 214-217)<br>3.2.2 "I suffer with postnatal depression, so that's why I like – she [GP] understands me as a mother and a person as well and there are a couple of other doctors that I like to see because they know the history" (P18, 173-176).
3.3 Access to health care	3.3.1 "It's nice to know that I can – if I do have any questions, I can phone up and speak to the nurses and they've always said, you know, no question is too silly, we are always at the end of the phone" (P7, 305-7)
3.4 Advice	3.4.1 "Spoke to me through what to do, the best thing to do, you know, keeping him cool, giving me plenty of information as well" (P19, 190-191)<br>3.4.2 "I think she probably said that we'd see some improvement within about four or five days" (P14, 222-3)
4. *Decision making*	
4.1Agreement/disagreement for an antibiotic prescription	4.1.1 "I must admit there was a little bit of relief – because it meant I hadn't overreacted and I think that she was poorly enough" (P3, 205-6).<br>4.1.2 "I think – I felt like she'd taken his illness seriously" (P10, 191).<br>4.1.3 "It felt like – good, you know something – you feel like something is being done to help the situation" (P4, 300-301).<br>4.1.4 "I know that he'd get better with them" (P20, 149)<br>4.1.5 "I was quite happy for her to have antibiotics to treat it because it was disturbing her sleep and showing no signs of dissipating by itself (P17, 35-36)<br>4.1.6 "…it was kind of expected really, neither happy nor unhappy" (P15, 153)<br>4.1.7 "But you don't see them every day, every night, how the kid's suffering or what they were doing, you know" (P12, 414-416)<br>4.1.8 "I was a bit shocked I thought, oh, right, okay, it's a cough – because obviously there's something not right, he's got a cough" (P8, 229-230)<br>4.1.9 "I think it was upsetting because I was thinking that my child would be suffering" (P10, 218-9).<br>4.1.10 "I think I would have been a little frustrated because at that point I did know that it was – it was getting worse and I probably would have thought – well it's going to need another trip to the doctor's in a couple of days – which is just a waste of time for me and I probably would have just been quite annoyed" (P9, 239-242)<br>4.1.11 "they'd sent me away saying she just had a virus" (P4, 68-9)<br>4.1.12 "…I wasn't convinced that they were right…really, because…if he's going to clear it up on his own, it's normally gone within three or four days […] But – yes, doctors know what they're talking about, don't they? You just go with it, don't you – really" (P16, 234)<br>4.1.13 "So, like I said, I go by the doctor's judgement to make it better for my

For peer review only - http://bmjopen.bmj.com/site/about/guidelines.xhtml

	daughter" (P1, 148-9)<br>4.1.14 "I'd probably let them tell me what's best for her, really. They are the professionals, I'm not" (P21, 216-217)<br>4.1.15 "in some cases you don't feel like you can challenge a doctor because they think well, they are the doctor, they're trained and if I start kicking off and making a fuss, they're just going to get really annoyed with me and just think I'm an overprotective parent" (P7, 278-281).<br>4.1.16 "I was here [GP surgery] yesterday, they told me there is no chest infection: why do I have to wait for another night for him to be treated? If he had been given [antibiotics] yesterday, they could – he could have been better, you know" (P12, 322-325)
4.2 Perspectives of GP's prescribing behaviour	4.2.1 "I'm of the generation that – you know – if you've got an infection, go to the doctor and get some antibiotics. I know now that – that things are very different" (P22, 349-351)<br>4.2.2 "Um…is where your body can't fight – you like become immune to the antibiotics and it doesn't do anything?" (P13, 455-456)<br>4.2.3 "Yes, overprescribing antibiotics or prescribing them for things like coughs and colds – which they are ineffective against anyway…So that can lead to superbugs" (P10, 376-7).<br>4.2.4 "No, never heard of it" (P11, 176)<br>4.2.5 "I think, in my experience, there's a good balance here – about prescribing them when they are needed" (P5, 431-432)<br>4.2.6 "I have heard of people going to the GP for really minor things and getting antibiotics and I find that frustrating" (P10, 233-234).<br>4.2.7 "There was a thing on the news the other day, that they're…doctors are going to earn more money if they prescribe less antibiotics or something. That's wrong, isn't it?" (P16, 110-111).
4.3 Parental knowledge of LRTI and their management	4.3.1 "you know instantly if your child is not right" (P3, 72)<br>4.3.2 "I know that they are going to – tell you to wait a week, so I wait a week before I go in" (P16, 255-6).<br>4.3.3 "three days is too much continuously, then I have to bring them in" (P12, 399-400)<br>4.3.4 "when they are getting worse and no sign of getting better, then I would, without doubt, go" (P5, 242-243)<br>4.3.5 "I feel so guilty to think – oh God – I feel like I'm wasting their time" (P8, 223)<br>4.3.6 "You sometimes feel a little bit like…a neurotic mother" (P4, 211)<br>4.3.7 "Yes, I know the signs: he has them so often that I can sort of pick the signs and as soon as that sign appears" (P11, 38-9)<br>4.3.8 "As a parent, you think the worst; you start Googling. I think – we knew it was some kind of chest infection" (P13, 152-4).<br>4.3.9 "I'm a nurse, so I kind of have quite a good understanding of what was going on" (P9, 71).<br>4.3.10 "I did think she had a cold and obviously until the said what it is, then I was obviously a bit shocked" (P1, 68-9)<br>4.3.11 "antibiotics shouldn't just be given out for coughs and colds and viruses and things…I know that GPs often say – antibiotics are no good for viruses, it's only bacterial infections" (P10, 246-7)<br>4.3.12 "because obviously a chest infection is obviously a viral infection, isn't it, so I think most infections to need something to help" (P1, 132-133)

For peer review only - http://bmjopen.bmj.com/site/about/guidelines.xhtml

BMJ Open

A qualitative interview study of parents' perspectives, concerns and experiences of the management of lower respiratory tract infections in children in primary care.

Journal:	*BMJ Open*
Manuscript ID	bmjopen-2016-015701.R1
Article Type:	Research
Date Submitted by the Author:	02-May-2017
Complete List of Authors:	Halls, Amy; University of Southampton, Primary Care and Population Sciences<br>van't Hoff, Catherine; Royal Bournemouth and Christchurch Hospitals NHS Foundation Trust<br>Little, Paul; University of Southampton, Primary Care and Population Science;<br>Verheij, Theo; University Medical Center Utrecht, Julius Center for Health Sciences and Primary Care<br>Leydon, Gerry; University of Southampton, Primary Care and Population Sciences
**Primary Subject Heading**:	General practice / Family practice
Secondary Subject Heading:	Qualitative research
Keywords:	PRIMARY CARE, QUALITATIVE RESEARCH, Antibiotics, Interviews

SCHOLARONE™ Manuscripts

For peer review only

Title: A qualitative interview study of parents' perspectives, concerns and experiences of the management of lower respiratory tract infections in children in primary care.

Halls, AV* * van't Hoff, CI** * Little, P* Verhejj, TJM.[+] Leydon, GM.*

*University of Southampton, Faculty of Medicine, Academic Unit of Primary Care and Population Sciences, Aldermoor Health Centre, Aldermoor Close, Southampton, S016 5ST, United Kingdom.

**Education Centre, Royal Bournemouth Hospital, Castle Lane East, Bournemouth, BH7 7DW, United Kingdom.

[+]Department of General Practice, UMC Utrecht, div. Julius Centrum, Huispost Str. 6.131, PO BOS 85500, 3508 GA Utrecht, The Netherlands

*The first two authors should be regarded as joint First Authors.

Corresponding author:
Dr Geraldine Leydon,
University of Southampton, Faculty of Medicine, Academic Unit of Primary Care and Population Sciences, Aldermoor Health Centre, Aldermoor Close, Southampton, S016 5ST, United Kingdom.
gerry@soton.ac.uk

Word count: 4309

For peer review only

Abstract

Objective: To explore parents' perspectives, concerns and experiences of the management of lower respiratory tract infections (LRTIs) in children in primary care.

Design: Qualitative semi-structured interview study.

Setting: UK primary care

Participants: 23 parents of children aged from six months to ten years presenting with LRTI in primary care.

Method: Thematic analysis of semi-structured interviews (either in person or by telephone) conducted with parents to explore their experiences and views on their children being prescribed antibiotics for LRTI.

Results: Four major themes were identified and these are perspectives on: 1) infection, 2) antibiotic use, 3) the GP appointment, and 4) decision-making around prescribing. Symptomatic relief was a key concern: the most troublesome symptoms were cough, breathing difficulty, fever and malaise. Many parents were reluctant to use self-care medication, tended to support antibiotic use, and believed they are effective for symptoms, illness duration and for preventing complications. However, parental expectations varied from a desire for reassurance and advice to an explicit preference for an antibiotic prescription. These preferences were shaped by: 1) the age of the child, with younger children perceived as more vulnerable because of their greater difficulty in communicating, and concerns about rapid deterioration; 2) the perceived severity of the illness; and 3) disruption to daily routine. When there was disagreement with the GP parents described feeling dismissed, and they were critical of inconsistent prescribing when they re-consult. When agreement between the parent and doctor featured, parents described a feeling of relief and legitimation for consulting, feeling reassured that the illness did indeed warrant a doctor's attention.

Conclusion: Symptomatic relief is a major concern for parents. Careful exploration of expectations, and eliciting worries about key symptoms and impact on daily life will be needed to help parents understand when a no antibiotic recommendation or delayed antibiotic recommendation is made.

For peer review only

Strengths and limitations of this study

- Provides a detailed view of key experiences and understandings of a sample of parents of children presenting with symptoms of LRTI in southern England.
- Only two fathers included (compared with 23 mothers) so the views of fathers were not fully captured.
- Semi-structured interviews were the optimal data collection method given the aims of the research and analytical saturation was reached.

For peer review only

For peer review only - http://bmjopen.bmj.com/site/about/guidelines.xhtml

BACKGROUND

The majority of respiratory tract infections (RTIs) are viral[1, 2] and will resolve spontaneously with analgesia and rest. Infections and treatment with antibiotics are important health concerns[3]. A third of children presenting to a GP with febrile illness receive an antibiotic prescription[4]. NICE (National Institute for Health and Care Excellence) guidance recommends patients be given no antibiotic prescription or a delayed prescription (to be fulfilled if symptoms worsen)[5], as well as advice about expected time course of infection and how to manage symptoms. Patients' education and general understanding of the medications they are prescribed affect compliance[6] and use of antibiotics leads to greater risk of development of antibiotic resistant bacteria[6, 7]. A US survey study of parents of children under 4 years suggested that parents often believed antibiotics were indicated for cough and upper respiratory tract infections[8], and in yet most parents in the US and UK are aware of antibiotic resistance and its complications[8, 9] or express concern about the overuse of antibiotics. More than half of adults may expect an antibiotic prescription when attending with symptoms of RTI.[6]

Uncomplicated acute lower respiratory tract infection is the commonest acute infection[10], and is likely to have the longest and highest symptom burden of all acute RTIs[11]. Previous work has assessed parents' understanding of the implications and indications for an antibiotic prescription[12] , with parents believing that antibiotics were for more severe illnesses, supported by GPs explanations. This study provides further insight into the tension between GPs and parents in their decision making, and explores key drivers of parental expectation and beliefs about antibiotics.

We report a qualitative study of the key concerns of parents about symptoms of their children presenting with acute LRTI in primary care and their perceptions about antibiotics and the consultation.

METHODS

Participants and procedure

Six research-active practices in the South West of England were identified through the Primary Care Research Network and a poster presentation by CvH at the Wessex Research Sites Initiative Conference. Practices searched their databases for children aged 6 months to 12 years who had presented to primary care with symptoms of LRTI between January 2013

For peer review only - http://bmjopen.bmj.com/site/about/guidelines.xhtml

and March 2015. The recruitment process is shown in Figure 1. A purposive sample was originally intended: however, due to initially slow recruitment, a convenience sampling strategy was used. Whilst this sampling approach can be limiting we recruited a good range of mothers in terms of age and occupation. Both urban and rural practices were invited to participate; however a deprivation scale was used to illustrate variation, as shown in Table 1. Parents were interviewed regardless of the method of GP consultation or whether antibiotics were prescribed.

Figure 1: Recruitment process

Table 1: Practice characteristics and recruitment

Practice	Total letters sent	Positive replies	Negative replies	Total replies	Number consented	Deprivation score*
1	33	1	2	3	1	13.5
2	106	3	1	3	2	19
3	44	3	0	3	2	22.8
4	85	9	3	12	9	7.1
5	12	2	0	2	3	13.6
6	120	7	6	12	6	13.3

******National General Practice Profiles* https://fingertips.phe.org.uk/profile/general-practice/data. **Higher score indicates a higher level of deprivation.**

Interviews

Two female interviewers (CvH and AH) conducted face to face interviews in the participant's home (n=20) and telephone interviews (n=3), each lasting approximately 30 to 60 minutes, with an average duration of 42 minutes. Both interviewers were aware of the NICE guidance regarding antibiotic prescribing for RTIs, and made field notes during interviews. All interviews were audio-recorded and transcribed verbatim. Qualitative interviews provided the best method for gathering insights into parents' views about their children's experiences of RTI and its management in primary care. The interview guide (Appendix 1) included key topic areas, not all of which are covered in this paper. Interviews explored parent views on

For peer review only - http://bmjopen.bmj.com/site/about/guidelines.xhtml

whether they would be willing to be randomised in a future study. Its semi-structured design gave flexibility to explore new areas if they arose.

Analysis

Inductive thematic analysis[13] was conducted on all transcripts to gain an understanding of the perspectives, attitudes and concerns of parents regarding the managing of LRTI in children in primary care. CvH and AH achieved familiarisation through repeated reading (and listening) of the transcripts. Patterns and themes in the data were identified by CvH and AH and labelled with codes: these code labels referred to the operationalisation of the theme content. A label and full description were provided for each theme. These codes and definitions were refined during a continuous process led by GL, CvH and AH: this involved themes being linked, grouped, moved, re-labelled, added and removed as appropriate in order to produce a set of themes, subthemes, and a coding manual, reviewed and confirmed by the full research team. The coding manual thoroughly explained all the data. .

Participants In total 23 participants took part in this study. In all interviews the mother of the child was interviewed, and fathers were present in two interviews, however anyone who fulfilled a caring role could have participated. The age of the mothers[1] ranged from 21 to 47 (median age 34) and the age of the children ranged from 6 months to 10 years (median age 1 year 9 months) (see Table 2 for participant characteristics).

Table 2: Participant characteristics

FINDINGS

Thematic analysis identified four themes relating to factors which enhanced our understanding of parents' perspectives, concerns and experiences of the management of LRTI in children in primary care. Table 3 outlines the themes and subthemes and these are used to structure the findings.

[1] The age of the fathers was not recorded.

For peer review only - http://bmjopen.bmj.com/site/about/guidelines.xhtml

For peer review only

Table 3: Themes identified in analysis

Themes	Sub-themes
1. Parental perspectives on infection	1.1 Symptoms<br>1.2 Previously similar infections<br>1.3 Parental concerns<br>1.4 Impact of child's illness<br>1.5 Home management strategies and medicine usage
2. Parental perspectives on antibiotic use for lower respiratory tract infection	2.1 Benefits and efficacy<br>2.2 Parental concerns
3. The GP appointment	3.1 Hopes and expectations<br>3.2 Positive/negative experiences<br>3.3 Access to health care<br>3.4 Advice
4. Decision making	4.1 Agreement/disagreement for an AB prescription<br>4.2 Perspectives of GP's prescribing behaviour<br>4.3 Parental knowledge of LRTI and their management

The following sections describe each theme in turn. Numbers in parentheses refer to the theme, subtheme, and relevant exemplary quotation provided in Appendix 2.

1. Parental perspectives on infection

Parental concerns: Parents spoke in detail about their concerns when their child was ill. By far the strongest and most frequent concern centred on breathing difficulties and airway limitations (1.1.1). Other concerns included general concerns or unhappiness that their child was unwell (1.1.2). However, some mothers were less concerned and this was associated with factors such as the child not being their first child, or if the child was perceived to be receiving appropriate treatment (1.1.3). Parents reported worry about symptoms their child had, including the duration of symptoms (1.3.4) or the qualitative difference in the child's behaviour (1.1.5).

For peer review only - http://bmjopen.bmj.com/site/about/guidelines.xhtml

Parents of younger children (two years and under) generally had more concerns due to their child's young age. These concerns centred on three areas: increased vulnerability (1.1.6); difficulties in communicating (1.1.7); and the potential for rapid deterioration (1.1.8). Some parents also expressed worry about complications (1.1.9).

Impact of child's illness: Parents were directly asked about the impact of the child's illness on them and their family and readily gave lack of sleep and tiredness across the family as the primary impact (1.2.1). Parents of children with underlying health conditions spoke in more detail about the impact of their child's condition (1.2.2). Working mothers spoke of social implications such as needing additional time off work and the subsequent financial impact (1.2.3). These pressures were also reflected when mothers talked about the need for antibiotics to shorten illness duration (see section 2.1).

Symptoms: All symptoms reported by parents are summarised in Table 4, which shows that the most commonly reported symptom by the study participants was a cough, but breathing, fever and malaise were also very commonly reported as troublesome symptoms.

Table 4: Reported symptoms

Symptoms	Frequency
Cough	18
Breathing difficulties	16
Fever/temperature	16
Malaise*	15
Difficulty sleeping	11
Feeding problems/appetite	10
Wheeze	10
Runny nose	9
Fatigue/tiredness	6
Vomit	6
Sore throat/hoarse voice	5
Ear ache	2
Pain	2

For peer review only - http://bmjopen.bmj.com/site/about/guidelines.xhtml

Headache	1
Fainting	1
Heart racing	1
Rash	1

*Malaise was used as a term to categorise anything parents said referring to a change in behaviours, character, or appearing unwell (1.1.1).

Previously similar infections: During the course of the interview parents recounted previous infections or the prolonged nature of the infection in question (1.2.1). Mothers of children with underlying health conditions often spoke of this in detail, but it is not central to the current study and so is not reported.

Home management strategies and medicine usage: Parents initially dealt with their child's infection in different ways. Parents reported trying home management strategies (see Table 5) or complementary medicines before visiting the GP (1.5.1). Parents' views on over-the-counter medicines were varied (1.5.2) although many parents used Calpol and Nurofen[2] to relieve symptoms, most commonly a temperature. Perhaps unsurprisingly many parents reported reluctance to use medications unless they felt it was strongly needed or recommended by a doctor (1.5.3).

Table 5: Home management strategies tried in addition to Calpol / Nurofen:

**Strategy**	**Interview mentioned in**
Steam inhalation	4,5,6,8,10,11,13,16
Vaporisers/scents	6,8,13,16
Throat pastilles/cough syrup	3,4,12,14,16,17
Drinks	3,4,8,12,19,21
Cold strategies	13,14,16
Raise the bed	8,16,17,19,23
Vicks	5,8,10,16, 21
Improved diet	5
Other*	2
No additional home management	1,7,9,15,20,22

*Participant 2's child took daily prophylactic antibiotics for a severe undiagnosed health condition: their home management strategy was to give a double dose, as recommended by the specialist.

For peer review only

[2] Analgesics widely available in the United Kingdom: Calpol contains paracetamol and Nurofen contains ibuprofen and codeine phosphate.

For peer review only - http://bmjopen.bmj.com/site/about/guidelines.xhtml

2. **Parental perspectives on antibiotic use for lower respiratory tract infection**

Parents spoke in detail about their perceptions of antibiotic use, their attitudes to prescribing and their concerns about antibiotics. Their views were varied but overall parents were happy with their experiences of antibiotic usage in their children.

Benefits and efficacy: Parents cited antibiotics as having three main benefits. Firstly, shortening illness duration (2.1.1), which had the additional benefit of helping parents to return to work (2.1.2). Secondly, antibiotics were described as being able to prevent complications (2.1.3). Thirdly, antibiotics were thought to provide symptomatic relief (2.1.4-2.1.5). Most parents expressed certainty that antibiotics were effective (2.1.6) and they often used their experience of their child recovering during past episodes to infer the effectiveness of antibiotics (2.1.7), although one parent did appreciate there were conditions in which antibiotics are more effective (2.1.8). In contrast, a minority of parents were aware that antibiotics are not always effective while some volunteered awareness of the lack of evidence for prescribing in children (2.1.9).

Parental concerns: Parental concerns about antibiotic use were predominantly about the composition of antibiotic medication (taste, texture, smell, and use of additives). Many parents commented negatively on the taste and texture whereas views on additives such as colourings were more varied. Generally parents had few concerns about adverse effects (2.2.1), especially when their child had not experienced any following previous use (2.2.2).

3. The GP appointment

Hopes and expectations: Parent interviews indicated varied hopes and expectations of the outcome of their GP appointments. Many parents described a desire for symptomatic relief, reassurance and / or advice (3.1.1, 3.1.2) while some, importantly in the minority, attended the GP consultation with an explicit wish for an antibiotic prescription (3.1.3).

Positive and negative experiences:

Many parents had positive views of the GP appointment; in particular when GPs were perceived to take time for a thorough assessment of the child (3.2.1) or with GPs who have built a history with the family (3.2.2). A physical examination has previously been shown to be a key expectation of parents[9]. However, some parents used the interviews as an

For peer review only - http://bmjopen.bmj.com/site/about/guidelines.xhtml

opportunity to voice frustrations over 'bad' appointments; these centred around feeling rushed, appointments running late or parental concerns not being taken seriously. Differences in parental and GP opinions also contributed to dissatisfaction (discussed further under theme 4).

Access to health care: Parents reported irritation about difficulties in getting appointments. They also discussed a lack of continuity of care, which could mean that deterioration was not noticed by the GP and parents had to recount their story on multiple occasions. Many spoke of needing to use out of hours services, and this brought with it new concerns around queueing, and seeing an unfamiliar doctor in an unfamiliar environment. One parent, living rurally, spoke of concerns around rapid deterioration out of normal working hours. However, many parents spoke positively of the use of the NHS 111 service and their advice. Parents of children with underlying health conditions spoke of easy access to medical expertise (3.3.1).

Advice: Parents were asked to discuss advice they were given at the index appointment. They primarily volunteered advice on home-management approaches they had already tried prior to the GP consultation (3.4.1). Many parents did not recall being given specific advice about the natural history of the illness. Of those interviewed some were asked to re-consult if there was no improvement and some recalled receiving advice on how long symptoms would last (3.4.2).

4. Decision making

A shared decision making process between parent and GP, or an explanation of why antibiotics were not necessary, usually led to a more acceptable consultation when parents did not explicitly express a desire for antibiotics.

GP-parent agreement and disagreement about the need for an antibiotic prescription: When there was agreement about the need for a prescription between GP and parents, many parents described a feeling of relief. A prescription seemed to give parents the feeling of illness legitimation[(1)](4.1.1, 4.1.2). In addition to this, parents reported feeling empowered by a prescription because it signalled a positive action taken to help their child (4.1.3). Parents also described that they were pleased because their child would now have symptomatic relief (4.1.4, 4.1.5). However, parents of children with frequent chest infections or underlying

For peer review only - http://bmjopen.bmj.com/site/about/guidelines.xhtml

health conditions did not seem to have strong opinions, viewing an antibiotic prescription as a necessary requirement (4.1.6).

When parents felt there was disagreement between them and their doctor, feelings of frustration were described. Frustration that doctors did not agree with their perception of the severity of infection (4.1.7), as there is a perceived link between the need for antibiotics only in severe infections[12],or frustration that doctors did not think antibiotics were indicated (4.1.8). Parents felt frustrated and upset at the lack of symptomatic relief or that they would need to re-consult (4.1.9, 4.1.10).

Parents spoke about feeling dismissed by their GP, especially if the infection was thought to be viral (4.1.11), as seen in previous studies[12]. Parents reported uncertainty when they disagreed with the doctor's decision, but this narrative was balanced by belief in ultimately trusting the doctor's judgement and their professional expertise (4.1.12-4.1.14).

Some parents described discomfort and the feeling they could not question the GP's judgement (4.1.15). The majority of parents trusted doctors' expertise but many parents recounted occasions when they had not been prescribed antibiotics initially and then had re-consulted shortly after and been given a prescription. By having to re-consult, parents felt doctors were not consistent, and that their persistence or pestering would result in a prescription. This often seemed to be closely related to dissatisfaction with the GP appointment (4.1.16). Interestingly, it is noted that GPs can feel increased pressure and are more likely to prescribe if a parent has re-consulted[14].

Perspective of GP's prescribing behaviour: Many parents had an awareness of overprescribing antibiotics and its consequences and some commented on doctors clearly trying to limit prescribing (4.2.1). Many parents understood antibiotic resistance to be an idea that over-use would result in your body becoming immune to the antibiotic; an idea which is echoed in the existing literature (4.2.2)[12, 15].

Parental concerns about over-use were reflected in their understanding of antibiotic resistance and superbugs. For example, parents with more accurate scientific knowledge often had a clear appreciation of the consequences of resistance (4.2.3). However, some parents were

For peer review only - http://bmjopen.bmj.com/site/about/guidelines.xhtml

quite clear that they had very limited understanding of the concept of antibiotic resistance (4.2.4).

Parents offered both positive and negative opinions on GP's prescribing behaviours (4.2.5). Negative opinions of prescribing behaviour were often from word of mouth. For example, a few parents spoke about factors they believed to influence prescribing (4.2.6) such as believing doctors earn more if they prescribe fewer antibiotics (4.2.7).

Parental understanding of LRTI and their management: Parents had a degree of prior knowledge regarding a suggested diagnosis, when to present, and if they felt antibiotics were indicated. Many parents spoke of knowing their child best (4.3.1), although this contrasted with previous comments, where parents described difficulties in communicating with young children. Parents appeared to have had different 'tipping points' with regard to help-seeking, such as when to present. Some parents tended to have a particular amount of time they were happy waiting before they consulted to help ensure they received antibiotics (i.e. illness duration would be such that a GP would be more likely to think antibiotics are required) (4.3.2). Others waited a specific number of days (4.3.3) or used a lack of improvement as a reason to consult (4.3.4). Although many parents had clear triggers for consulting, they simultaneously reported concerns about wasting doctors' time (4.3.5), or appearing over-anxious (4.3.6).

Parents often described having a good idea of the diagnosis before presenting. Parental knowledge of a diagnosis came from a variety of experiences: knowing their child, and prior experience, was a chief way of gauging severity and the need to consult (4.3.7). Other parents had conducted internet research to find a diagnosis (4.3.8). Two parents were health care professionals, and relied on their professional expertise (4.3.9) whereas some parents described not having an idea of the likely diagnosis (4.3.10).

There was some confusion over the role of antibiotics in treating LRTI. Some parents understood that antibiotics were only indicated for bacterial infections (4.3.11), whilst others were unclear of the difference between bacterial and viral infections and did not know when antibiotics were indicated (4.3.12), as seen in the current literature[12].

DISCUSSION

For peer review only - http://bmjopen.bmj.com/site/about/guidelines.xhtml

The study identified four themes that related to parents' perspectives, attitudes and concerns regarding the management of LTRI in children in primary care.

Main findings

Overall, this study provides insight into the views of parents of children who have consulted with their GP for suspected LRTI. It presents up to date evidence of the most significant concerns for parents, primarily sudden deterioration, breathing difficulties or the child's young age. Whilst most believed that antibiotics were effective for the symptoms of LRTI, many parents did not present with a certain expectation for antibiotics, and yet many felt frustration if the GP did not agree with their perception of the need to actively treat their child. There was a tension between parents wanting symptomatic relief for their individual child, the doctor's expertise and their inclination to prescribe based on their understanding of need in the population in general.

When asked, many parents did not recall being given specific advice on the infection duration. However, interviews revealed that parents did have some understanding about likely duration of a LRTI as shown through the 'tipping points' discussed previously. NICE guidance[5] advises patients should be advised about the natural history of RTIs including usual total length. Interviews suggest this is important for GPs to emphasise so parents can be reassured about when to present.

Comparison with existing literature

Clinicians prescribe for medical indication and other reasons such as perceived pressure from parents[1, 14] but this pressure is not always evident. Inappropriate antibiotic prescriptions may result in problems such as antimicrobial resistance[3, 12] as well as the medicalisation of a self-limiting illness[14, 16]. Whilst this study showed many parents wanted antibiotics, some indicated that they were happy to withhold antibiotic treatment and continue with non-medical self-management strategies and over the counter medicines in a context of reassurance, and when given a sense of illness legitimation. Parental satisfaction was likely to be highest when the GP was perceived to be thorough, that they were listened to and had their concerns taken seriously. Where appropriate, having the medical history of the child or family taken into account also helped with parental satisfaction[14].

For peer review only - http://bmjopen.bmj.com/site/about/guidelines.xhtml

This study shows that parents often waited and consulted when they had reached a 'tipping point' at which they felt the severity and duration of the child's illness was such that some action in the form of prescribing antibiotics was warranted. This resonates more generally with research into urinary tract infections[17] in which respondents described an initial reluctance to consult. However, a visit was eventually prompted by symptom severity, often in conjunction with duration. This idea is echoed in a research assessing clinicians' prescribing practice[14]: in the uncertainty of whether this child needs antibiotics, clinicians have their own tipping point. This could be influenced by factors such as whether the parent has previously consulted, proximity to the weekend, or concerns that the parent would not recognise a clinical deterioration. Corroborating evidence with this study lends credibility to our research, adding to this important area in which we need to build a rich understanding. Previous research has identified a need to share knowledge with patients to ensure clear understanding of antibiotics and their appropriate use[12, 15]. However, this present study also showed that even if parents had a good understanding of LRTI and its causes, the perceived vulnerability of their child could influence them to present sooner than they may have done had the child been older and more able to communicate how they were feeling.

IMPLICATIONS FOR CLINICAL PRACTICE

Overall, most parents were satisfied with their child's GP consultation. Higher satisfaction was evident in parent narratives when parents believed their concerns about their child's health had been taken seriously, from a thorough examination to having more time spent with them. However, the tension between the parent and GP remains evident[12]. Increased GP sensitivity to the wider concerns of parents and the need to employ communication strategies that encourage parental participation and validation of their concerns could ease the path towards improved parent/patient and GP antibiotic discussions and ultimately towards more prudent antibiotic prescribing and use.

The interviews showed that whilst some parents did present to their GP with the expectation of receiving an antibiotic prescription, many parents did not: other aims for the consultation included illness legitimisation and advice for symptomatic relief using home management strategies. GPs need to be aware that parents are often willing to accept a treatment other than antibiotics, and often would rather have an alternative non-antibiotic approach. Providing structured information and exploration of concerns can help negotiate lower

For peer review only - http://bmjopen.bmj.com/site/about/guidelines.xhtml

antibiotic use[18)] as it does among adults[19]. Parental willingness to consider alternative management strategies often linked with the knowledge parents had about the causes of LRTI and appropriate treatment. Across the interviews, parents displayed varying levels of understanding and beliefs about causes, duration and treatment of LRTI and when they should present. Clarifying patient information, and helping to enhance general understanding, could help to reduce unnecessary GP consultations and smooth the interactional path when parents do consult.

STRENGTHS AND LIMITATIONS

The sample of participants is not representative of the wider population and just sensitises us to key experiences and understandings of a sample of parents. Participants may have had stronger opinions or other reasons for being interviewed than 'typical' or non-participant parents. Only two fathers were included in interviews (compared with 23 mothers) so fathers' views were not fully captured in this study. Interviews occurred at varied times in relation to the infection/index consultation for the consulting 'child', which means that some parents had differing lengths of recall, although for most it was only a matter of weeks. Finally, interviews provide insight into perspectives on events not a direct window to the event themselves (such as the GP appointment). However, semi-structured interviews were the optimal data collection method given the aims of this research project.

CONCLUSION

This study aimed to explore factors important to parents in the management of LTRI in children in primary care. Parental views were varied, but clearly indicated that symptomatic relief is a key consideration for their decision-making regarding when to consult and management preferences. Parents do not necessarily expect an antibiotic prescription and a satisfactory consultation can be achieved by being perceived as thorough and in legitimating parents' reason for consulting. There is an on-going need for GPs to explore concerns and expectations carefully, and to tailor advice, information and reassurance for parents - particularly addressing the natural history, worrying symptoms, and the likely impact of antibiotics on symptom severity, duration and complications.

Acknowledgments

For peer review only - http://bmjopen.bmj.com/site/about/guidelines.xhtml

Interviewees for their invaluable contributions on this important topic.

NIHR Fellowship funding for Geraldine M Leydon during the conduct of this research.

Ethical approval

Ethical approval for this study was granted by Wales REC 6 reference 14/WA/1226.

Funding

This project was funded by the National Institute for Health Research Health Technology Assessment Programme (project number 13/34/64).

Department of Heath disclaimer:

The views and opinions expressed therein are those of the authors and do not necessarily reflect those of the Health Technology Assessment Programme, NIHR, NHS or the Department of Health.

No further data are available.

There are no competing interests.

Contributors:

GML was responsible for all aspects of the study including design, data analysis and interpretation, and manuscript preparation. AH and CvH collected, analysed and interpreted the data, and prepared the manuscript led by GL. PL is Principal Investigator of a larger affiliated study Antibiotics for lower Respiratory Tract Infection in Children presenting in Primary Care (ARTIC PC) and PL and TV were involved in the generation of the study idea and design, and manuscript comments and revisions.

References:

1. Peters S, Rowbotham S, Chisholm A, Wearden A, Moschogianis S, Cordingley L, et al. Managing self-limiting respiratory tract infections: a qualitative study of the usefulness of the delayed prescribing strategy. Br J Gen Pract. 2011;61(590):e579-89.

2. Thompson PL, Spyridis N, Sharland M, Gilbert RE, Saxena S, Long PF, et al. Changes in clinical indications for community antibiotic prescribing for children in the UK from 1996 to 2006: will the

For peer review only - http://bmjopen.bmj.com/site/about/guidelines.xhtml

new NICE prescribing guidance on upper respiratory tract infections just be ignored? Arch Dis Child. 2009;94(5):337-40.
3. Davies S, Fowler T, Watson J, Livermore D, Walker D. Annual Report of the Chief Medical Officer: infection and the rise of antimicrobial resistance. Editorial Material. London: Department of Health; 2011 MAY 11 2013. Report No.: 0140-6736 Contract No.: 9878.
4. Moore M. Antibiotics: time to act. British Journal of General Practice. 2013;63(612):340-1.
5. National Institute for Health and Clinical Excellence: Guidance. Respiratory Tract Infections - Antibiotic Prescribing: Prescribing of Antibiotics for Self-Limiting Respiratory Tract Infections in Adults and Children in Primary Care. London: National Institute for Health and Clinical Excellence (UK); 2008.
6. McNulty CA, Nichols T, French DP, Joshi P, Butler CC. Expectations for consultations and antibiotics for respiratory tract infection in primary care: the RTI clinical iceberg. Br J Gen Pract. 2013;63(612):e429-36.
7. Little P, Stuart B, Moore M, Coenen S, Butler CC, Godycki-Cwirko M, et al. Amoxicillin for acute lower-respiratory-tract infection in primary care when pneumonia is not suspected: a 12-country, randomised, placebo-controlled trial. Lancet Infect Dis. 2013;13(2):123-9.
8. Trepka MJ, Belongia EA, Chyou PH, Davis JP, Schwartz B. The effect of a community intervention trial on parental knowledge and awareness of antibiotic resistance and appropriate antibiotic use in children. Pediatrics. 2001;107(1):E6.
9. Francis N, Phillips R, Wood F, Hood K, Simpson S, Butler C. Parents' and clinicians' views of an interactive booklet about respiratory tract infections in children: a qualitative process evaluation of the EQUIP randomised controlled trial. Bmc Family Practice. 2013;14.
10. Gulliford MC, Dregan A, Moore MV, Ashworth M, Staa T, McCann G, et al. Continued high rates of antibiotic prescribing to adults with respiratory tract infection: survey of 568 UK general practices. BMJ Open. 2014;4(10):e006245.
11. Thompson M, Vodicka TA, Blair PS, Buckley DI, Heneghan C, Hay AD, et al. Duration of symptoms of respiratory tract infections in children: systematic review. BMJ. 2013;347:f7027.
12. Cabral C, Ingram J, Lucas PJ, Redmond NM, Kai J, Hay AD, et al. Influence of Clinical Communication on Parents' Antibiotic Expectations for Children With Respiratory Tract Infections. Annals of family medicine. 2016;14(2):141-7.
13. Braun V, Clarke V. Using thematic analysis in psychology. Qualitative Research in Psychology [Internet]. 2006 05.01.15; 3(2):[77-101 pp.].
14. Horwood J, Cabral C, Hay AD, Ingram J. Primary care clinician antibiotic prescribing decisions in consultations for children with RTIs: a qualitative interview study. The British journal of general practice : the journal of the Royal College of General Practitioners. 2016;66(644):e207-13.
15. Brookes-Howell L, Elwyn G, Hood K, Wood F, Cooper L, Goossens H, et al. 'The Body Gets Used to Them': Patients' Interpretations of Antibiotic Resistance and the Implications for Containment Strategies. Journal of General Internal Medicine. 2012;27(7):766-72.
16. Andrews T, Thompson M, Buckley DI, Heneghan C, Deyo R, Redmond N, et al. Interventions to Influence Consulting and Antibiotic Use for Acute Respiratory Tract Infections in Children: A Systematic Review and Meta-Analysis. Plos One. 2012;7(1).
17. Leydon GM, Turner S, Smith H, Little P, team U. Women's views about management and cause of urinary tract infection: qualitative interview study. BMJ. 2010;340:c279.
18. Francis NA, Butler CC, Hood K, Simpson S, Wood F, Nuttall J. Effect of using an interactive booklet about childhood respiratory tract infections in primary care consultations on reconsulting and antibiotic prescribing: a cluster randomised controlled trial. British Medical Journal. 2009;339.
19. Little P, Stuart B, Francis N, Douglas E, Tonkin-Crine S, Anthierens S, et al. Effects of internet-based training on antibiotic prescribing rates for acute respiratory-tract infections: a multinational, cluster, randomised, factorial, controlled trial. Lancet. 2013;382(9899):1175-82.

For peer review only - http://bmjopen.bmj.com/site/about/guidelines.xhtml

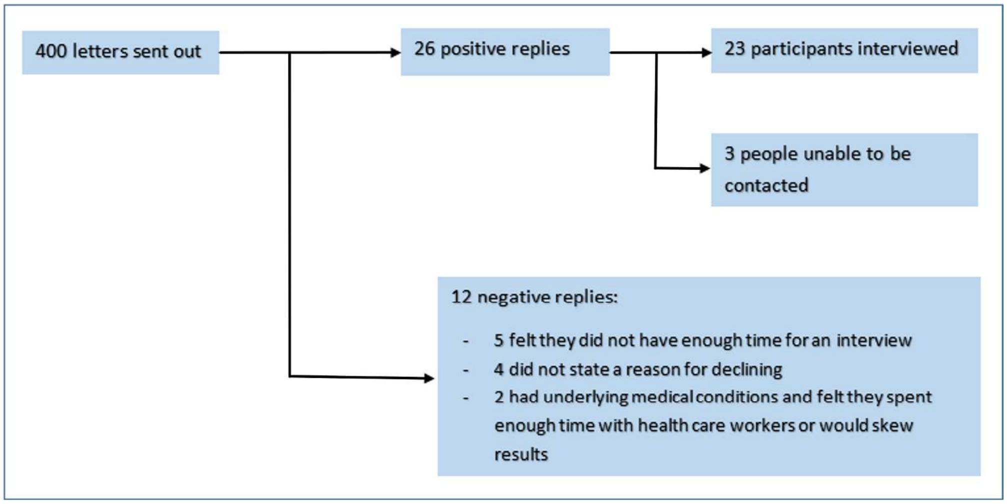

Recruitment process

203x100mm (300 x 300 DPI)

Appendix 1: Interview guide

Section 1: presenting illness

- Before visiting the GP can you describe what happened/when you first noticed [name] was poorly?
- By the time you took [name] to the GP what particular symptoms did [name] have?
    - What, timings, together/separate?
- How did you feel about [name] being ill at the time?
- What was it that made you take [name] to the GP?
    - What was most concerning about the illness?
        - Eg compared to other coughs/illness
    - Had they had any previously similar episodes?
        - Generally quite well?
- Can you describe what you thought might be wrong with [name] at the time?
    - Read anything on the internet
    - Advice from friends/family/pharmacist
- How were you feeling in yourself?
    - Did [name's] illness impact on the family?
    - Concerns re cross-infection?
- Before visiting the GP had you tried any form of treatment?
    - E.g Calpol, Nurofen?
    - Talk to other parents?
    - Generally do you wait and see/get medicines?
- What happened at your appointment?
    - What did the GP do?
    - Did they give you any medicines/advice (and if so what) /examine [name]?
- What did you hope to get from the GP appointment?
    - Reassure, prescribe antibiotics
- Can you think back and describe whether your hopes/expectations about the GP consultation were met?
    - Why / why not?
- If [name] had the same symptoms again how would you manage the situation next time?
    - Would you do anything differently?
        - Visit GP sooner?
        - Delay?

For peer review only - http://bmjopen.bmj.com/site/about/guidelines.xhtml

- Not attend?
- Make wishes more explicit with GP?
- Reconsider value of Abs?

Section 2: understanding of antibiotics

- [Name] was/wasn't prescribed a/bs, how did you feel about that?
  - Why did you think a/bs were/were not needed?
  - E.g. Did you want [name] to have antibiotics?
  - How did antibiotics help [name] and your family get better?
    - Quicker recovery
    - Feel empowered
    - Could go back to school/playgroup?
  - How would you have felt if [name] had not received antibiotics?
    - Can you think of a time you have taken [name] to the GP and they weren't given a/bs?
    - Do you think the a/bs made [name] better?
    - Would they have got better themselves with no a/bs?
- Can you tell me about your thoughts about antibiotics and their use in children with chest infections?
  - E.g. effective/not-effective? Best avoided?
  - E.g. any concerns about giving antibiotics to children?
  - Side effects
  - Read anything of the Internet etc?
  - Concerns about sugar content/flavourings/colouring?
  - Taste issues?
- Have you heard of antibiotic resistance?
  - What does it mean to you?
  - Check understanding re resistant bacteria vs resistant body
- Have you heard about 'super-bugs'? Do you see this to be linked to prescribing antibiotics?
- Was it explained to you that you must finish all the antibiotics prescribed? And why it is important?

- [Remind parents that it does not matter if they are unsure, and provide information leaflet explaining antibiotic resistance after the interview.]

For peer review only - http://bmjopen.bmj.com/site/about/guidelines.xhtml

Recruitment/participation:

- A future trial is currently being developed to test how effective antibiotics are because we need to know which antibiotics work best in which children. We know that whilst most children get antibiotics, many children will probably settle without antibiotics but some will benefit from antibiotics. What we don't know is if antibiotics help or not and which children get benefit from antibiotics and which children suffer side effects (rashes, diarrhoea, resistance).
- The team are going to be doing a trial which would involve some children taking the medicine and others taking a dummy/sugar-coated pill, this would be randomly allocated. All the children will be fully monitored during the trial and will be carefully reviewed if they are not improving.
    - If you had the opportunity would you be interested in participating in this kind of trial?
    - What do you think about the possibility of your child taking the dummy pill?
- How did you feel about being contacted by letter?
    - Preferred GP/face to face?

For peer review only - http://bmjopen.bmj.com/site/about/guidelines.xhtml

Appendix 2: Exemplary Quotations

Themes	Illustrative quotations from participants
1. Parental perspectives on infection	
1.1 Parental concerns	1.1.1 "Oh, terrified. Because of the breathing, I was really frightened about – because the coughing – when she's having these coughing fits – it's just really scary and I was – I was scared to put her to bed because of the wheezing" (P4, 106-108)<br>1.1.2 "all you want to do is just stop the cough and you can't" (P21, 83)<br>1.1.3 "Fine, obviously just a little bit concerned, that was all; but apart from that, I was fine – as long as she was getting the treatment that she needed, then I was happy" (P1, 71-72)<br>1.1.4 "she'd been unwell really for the biggest part of the beginning of the week" (P22, 19)<br>1.1.5 "I think my main concern was the fact that he wasn't his usual – playful self" (P9, 34)<br>1.1.6 "where it's – such a small body and it's so hard for them to actually fight back" (P10, 142-3)<br>1.1.7 "It's – the problem we have with [name] is it's very hard to know when he is unwell because he just carries on and when – where he's quite young as well – he won't sit there and say, mummy my throat hurts, he'll just carry on as normal" (P7, 30-32)<br>1.1.8. "You know, because he's only little as well and at that age – because they can go downhill very quickly, that's what I was worried about" (P8, 89-90).<br>1.1.9 "there was that fear that it could have – it could be developing something worse" (P13, 302-303)
1.2 Impact of child's illness	1.2.1 "[son] did hear her coughing, so it did keep him awake as well" (P21, 313-314)<br>1.2.2 "she gets very out of breath very quickly, so she can't join in her sports days, things like that; she doesn't ever complete a full week at pre-school" (P2, 109-110)<br>1.2.3 "a lot of times parents are working, both parents, you need to get back to work; it costs money to have a child off sick and then you have to stay off sick, you lose a day's pay or – or perhaps you have to pay a childminder, even though you're not at work, you have to stay home from work as well" (P10, 286-289)
1.3 Symptoms	1.3.1 "He wasn't his usual playful self, he was just sort of a bit off colour" (P9, 21-22)
1.4 Previously similar infections	1.4.1 "Well she's had – antibiotics now for chest infections probably three or four times" (P14, 10)
2. Parental perspectives on antibiotic use for lower respiratory tract infection	
2.1 Benefits and efficacy	2.1.1 "If I didn't give her the antibiotics, I think maybe she would of, but it probably would have tooken a lot, lot longer, but…no, I think the antibiotics helped. They helped – and they make it quicker as well, so they are not so ill for long" (P1, 173-175)<br>2.1.2 "anything you can do to help speed up the process and get her better, so that you can get back to normal, because it's – it has such an impact" (P4, 327-329)<br>2.1.3 "I think the risks of something bad happening or, you know, in the night and not being able to breathe – would…yes, would have been more worrying and, you know maybe either I would have had to have called an ambulance or taken him into hospital or something like that. And I think that was definitely avoided by the antibiotics" (P5, 284-289)<br>2.1.4 "If there's antibiotics that can fix whatever they've got then – yes – that they can sleep better and breath" (P5, 193-4)<br>2.1.5 "I don't think there's any point in letting a little baby cough like that and suffer like that for – I don't know how long – I – worry that they won't – not that they won't get over eventually themselves, but – I think..that it's my job to keep them well and – give them – yes – the opportunity to get better" (P5, 202-5)

For peer review only - http://bmjopen.bmj.com/site/about/guidelines.xhtml

	2.1.6 "They 100% work, 100%" (P1, 231)<br>2.1.7 "I mean they made [name] better in December, so, yes, they must be effective in children" (P3, 280-281)<br>2.1.8 "I do, yes. Antibiotics certainly helped both my children – but I think they are only effective if you use them correctly, so if you do finish the whole course of treatment" (P10, 275-276)<br>2.1.9 "should he be having antibiotics, you know – is it going to make any difference at all?" (P9, 85-86)
2.2 Parental concerns about problems with antibiotics	2.2.1 "no, upset like nasty nappies and things but – no – nothing, aside from that" (P18, 314-315).<br>2.2.2 "no, none that I'm aware of" (P23, 161)
3. *The GP appointment*	
3.1 Hopes and expectations	3.1.1 "Just to get her better really" (P21, 179)<br>3.1.2 "just that I wasn't a neurotic mother, confirmation that there was something wrong with my child and something to fix her" (P17, 125-6).<br>3.1.3 "I was hoping for antibiotics. I was – I kind of knew that it was going to be antibiotics, again, just going from experience" (P7, 180-1).
3.2 Positive / negative experiences	3.2.1 "the lady we saw the next day was lovely; she really took the time to…you know, really – sort of – looked at everything and – seemed, you know, genuine, genuinely – sort of concerned for her and what have you" (P4, 214-217)<br>3.2.2 "I suffer with postnatal depression, so that's why I like – she [GP] understands me as a mother and a person as well and there are a couple of other doctors that I like to see because they know the history" (P18, 173-176).
3.3 Access to health care	3.3.1 "It's nice to know that I can – if I do have any questions, I can phone up and speak to the nurses and they've always said, you know, no question is too silly, we are always at the end of the phone" (P7, 305-7)
3.4 Advice	3.4.1 "Spoke to me through what to do, the best thing to do, you know, keeping him cool, giving me plenty of information as well" (P19, 190-191)<br>3.4.2 "I think she probably said that we'd see some improvement within about four or five days" (P14, 222-3)
4. *Decision making*	
4.1Agreement/disagreement for an antibiotic prescription	4.1.1 "I must admit there was a little bit of relief – because it meant I hadn't overreacted and I think that she was poorly enough" (P3, 205-6).<br>4.1.2 "I think – I felt like she'd taken his illness seriously" (P10, 191).<br>4.1.3 "It felt like – good, you know something – you feel like something is being done to help the situation" (P4, 300-301).<br>4.1.4 "I know that he'd get better with them" (P20, 149)<br>4.1.5 "I was quite happy for her to have antibiotics to treat it because it was disturbing her sleep and showing no signs of dissipating by itself (P17, 35-36)<br>4.1.6 "…it was kind of expected really, neither happy nor unhappy" (P15, 153)<br>4.1.7 "But you don't see them every day, every night, how the kid's suffering or what they were doing, you know" (P12, 414-416)<br>4.1.8 "I was a bit shocked I thought, oh, right, okay, it's a cough – because obviously there's something not right, he's got a cough" (P8, 229-230)<br>4.1.9 "I think it was upsetting because I was thinking that my child would be suffering" (P10, 218-9).<br>4.1.10 "I think I would have been a little frustrated because at that point I did know that it was – it was getting worse and I probably would have thought – well it's going to need another trip to the doctor's in a couple of days – which is just a waste of time for me and I probably would have just been quite annoyed" (P9, 239-242)<br>4.1.11 "they'd sent me away saying she just had a virus" (P4, 68-9)<br>4.1.12 "…I wasn't convinced that they were right…really, because…if he's going to clear it up on his own, it's normally gone within three or four days […] But – yes, doctors know what they're talking about, don't they? You just go with it, don't you – really" (P16, 234)<br>4.1.13 "So, like I said, I go by the doctor's judgement to make it better for my daughter" (P1, 148-9)

For peer review only - http://bmjopen.bmj.com/site/about/guidelines.xhtml

	4.1.14 "I'd probably let them tell me what's best for her, really. They are the professionals, I'm not" (P21, 216-217)<br>4.1.15 "in some cases you don't feel like you can challenge a doctor because they think well, they are the doctor, they're trained and if I start kicking off and making a fuss, they're just going to get really annoyed with me and just think I'm an overprotective parent" (P7, 278-281).<br>4.1.16 "I was here [GP surgery] yesterday, they told me there is no chest infection: why do I have to wait for another night for him to be treated? If he had been given [antibiotics] yesterday, they could – he could have been better, you know" (P12, 322-325)
4.2 Perspectives of GP's prescribing behaviour	4.2.1 "I'm of the generation that – you know – if you've got an infection, go to the doctor and get some antibiotics. I know now that – that things are very different" (P22, 349-351)<br>4.2.2 "Um…is where your body can't fight – you like become immune to the antibiotics and it doesn't do anything?" (P13, 455-456)<br>4.2.3 "Yes, overprescribing antibiotics or prescribing them for things like coughs and colds – which they are ineffective against anyway…So that can lead to superbugs" (P10, 376-7).<br>4.2.4 "No, never heard of it" (P11, 176)<br>4.2.5 "I think, in my experience, there's a good balance here – about prescribing them when they are needed" (P5, 431-432)<br>4.2.6 "I have heard of people going to the GP for really minor things and getting antibiotics and I find that frustrating" (P10, 233-234).<br>4.2.7 "There was a thing on the news the other day, that they're…doctors are going to earn more money if they prescribe less antibiotics or something. That's wrong, isn't it?" (P16, 110-111).
4.3 Parental knowledge of LRTI and their management	4.3.1 "you know instantly if your child is not right" (P3, 72)<br>4.3.2 "I know that they are going to – tell you to wait a week, so I wait a week before I go in" (P16, 255-6).<br>4.3.3 "three days is too much continuously, then I have to bring them in" (P12, 399-400)<br>4.3.4 "when they are getting worse and no sign of getting better, then I would, without doubt, go" (P5, 242-243)<br>4.3.5 "I feel so guilty to think – oh God – I feel like I'm wasting their time" (P8, 223)<br>4.3.6 "You sometimes feel a little bit like…a neurotic mother" (P4, 211)<br>4.3.7 "Yes, I know the signs: he has them so often that I can sort of pick the signs and as soon as that sign appears" (P11, 38-9)<br>4.3.8 "As a parent, you think the worst; you start Googling. I think – we knew it was some kind of chest infection" (P13, 152-4).<br>4.3.9 "I'm a nurse, so I kind of have quite a good understanding of what was going on" (P9, 71).<br>4.3.10 "I did think she had a cold and obviously until the said what it is, then I was obviously a bit shocked" (P1, 68-9)<br>4.3.11 "antibiotics shouldn't just be given out for coughs and colds and viruses and things…I know that GPs often say – antibiotics are no good for viruses, it's only bacterial infections" (P10, 246-7)<br>4.3.12 "because obviously a chest infection is obviously a viral infection, isn't it, so I think most infections to need something to help" (P1, 132-133)

For peer review only - http://bmjopen.bmj.com/site/about/guidelines.xhtml

COREQ (COnsolidated criteria for REporting Qualitative research) Checklist

A checklist of items that should be included in reports of qualitative research. You must report the page number in your manuscript where you consider each of the items listed in this checklist. If you have not included this information, either revise your manuscript accordingly before submitting or note N/A.

Topic	Item No.	Guide Questions/Description	Reported on Page No.
**Domain 1: Research team and reflexivity**			
Personal characteristics			
Interviewer/facilitator	1	Which author/s conducted the interview or focus group?	5
Credentials	2	What were the researcher's credentials? E.g. PhD, MD	Author bios
Occupation	3	What was their occupation at the time of the study?	
Gender	4	Was the researcher male or female?	5
Experience and training	5	What experience or training did the researcher have?	Not reported
Relationship with participants			
Relationship established	6	Was a relationship established prior to study commencement?	NA
Participant knowledge of the interviewer	7	What did the participants know about the researcher? e.g. personal goals, reasons for doing the research	NA
Interviewer characteristics	8	What characteristics were reported about the inter viewer/facilitator? e.g. Bias, assumptions, reasons and interests in the research topic	Not reported
**Domain 2: Study design**			
Theoretical framework			
Methodological orientation and Theory	9	What methodological orientation was stated to underpin the study? e.g. grounded theory, discourse analysis, ethnography, phenomenology, content analysis	
Participant selection			
Sampling	10	How were participants selected? e.g. purposive, convenience, consecutive, snowball	5
Method of approach	11	How were participants approached? e.g. face-to-face, telephone, mail, email	5
Sample size	12	How many participants were in the study?	5
Non-participation	13	How many people refused to participate or dropped out? Reasons?	5
Setting			
Setting of data collection	14	Where was the data collected? e.g. home, clinic, workplace	6
Presence of non-participants	15	Was anyone else present besides the participants and researchers?	5
Description of sample	16	What are the important characteristics of the sample? e.g. demographic data, date	7
Data collection			
Interview guide	17	Were questions, prompts, guides provided by the authors? Was it pilot tested?	20
Repeat interviews	18	Were repeat inter views carried out? If yes, how many?	NA
Audio/visual recording	19	Did the research use audio or visual recording to collect the data?	6
Field notes	20	Were field notes made during and/or after the inter view or focus group?	5
Duration	21	What was the duration of the inter views or focus group?	6
Data saturation	22	Was data saturation discussed?	3
Transcripts returned	23	Were transcripts returned to participants for comment and/or	NA

For peer review only

Topic	Item No.	Guide Questions/Description	Reported on Page No.
		correction?	
**Domain 3: analysis and findings**			
Data analysis			
Number of data coders	24	How many data coders coded the data?	6
Description of the coding tree	25	Did authors provide a description of the coding tree?	No
Derivation of themes	26	Were themes identified in advance or derived from the data?	6
Software	27	What software, if applicable, was used to manage the data?	NA
Participant checking	28	Did participants provide feedback on the findings?	NA
Reporting			
Quotations presented	29	Were participant quotations presented to illustrate the themes/findings? Was each quotation identified? e.g. participant number	23
Data and findings consistent	30	Was there consistency between the data presented and the findings?	8
Clarity of major themes	31	Were major themes clearly presented in the findings?	8
Clarity of minor themes	32	Is there a description of diverse cases or discussion of minor themes?	8

Developed from: Tong A, Sainsbury P, Craig J. Consolidated criteria for reporting qualitative research (COREQ): a 32-item checklist for interviews and focus groups. *International Journal for Quality in Health Care*. 2007. Volume 19, Number 6: pp. 349 – 357

Once you have completed this checklist, please save a copy and upload it as part of your submission. DO NOT include this checklist as part of the main manuscript document. It must be uploaded as a separate file.

For peer review only

BMJ Open

A qualitative interview study of parents’ perspectives, concerns and experiences of the management of lower respiratory tract infections in children in primary care.

Journal:	*BMJ Open*
Manuscript ID	bmjopen-2016-015701.R2
Article Type:	Research
Date Submitted by the Author:	18-Jul-2017
Complete List of Authors:	Halls, Amy; University of Southampton, Primary Care and Population Sciences<br>van't Hoff, Catherine; Royal Bournemouth and Christchurch Hospitals NHS Foundation Trust<br>Little, Paul; University of Southampton, Primary Care and Population Science;<br>Verheij, Theo; University Medical Center Utrecht, Julius Center for Health Sciences and Primary Care<br>Leydon, Gerry; University of Southampton, Primary Care and Population Sciences
**Primary Subject Heading**:	General practice / Family practice
Secondary Subject Heading:	Qualitative research
Keywords:	PRIMARY CARE, QUALITATIVE RESEARCH, Antibiotics, Interviews

SCHOLARONE™ Manuscripts

For peer review only

Title: A qualitative interview study of parents' perspectives, concerns and experiences of the management of lower respiratory tract infections in children in primary care.

Halls, AV* * van't Hoff, CI** * Little, P* Verheij, TJM.[+] Leydon, GM.*

*University of Southampton, Faculty of Medicine, Academic Unit of Primary Care and Population Sciences, Aldermoor Health Centre, Aldermoor Close, Southampton, S016 5ST, United Kingdom.

**Education Centre, Royal Bournemouth Hospital, Castle Lane East, Bournemouth, BH7 7DW, United Kingdom.

[+]Department of General Practice, UMC Utrecht, div. Julius Centrum, Huispost Str. 6.131, PO BOS 85500, 3508 GA Utrecht, The Netherlands

*The first two authors should be regarded as joint First Authors.

Corresponding author:
Dr Geraldine Leydon,
University of Southampton, Faculty of Medicine, Academic Unit of Primary Care and Population Sciences, Aldermoor Health Centre, Aldermoor Close, Southampton, S016 5ST, United Kingdom.
gerry@soton.ac.uk

Word count: 4309

For peer review only - http://bmjopen.bmj.com/site/about/guidelines.xhtml

Abstract

Objective: To explore parents' perspectives, concerns and experiences of the management of lower respiratory tract infections (LRTIs) in children in primary care.

Design: Qualitative semi-structured interview study.

Setting: UK primary care

Participants: 23 parents of children aged from six months to ten years presenting with LRTI in primary care.

Method: Thematic analysis of semi-structured interviews (either in person or by telephone) conducted with parents to explore their experiences and views on their children being prescribed antibiotics for LRTI.

Results: Four major themes were identified and these are perspectives on: 1) infection, 2) antibiotic use, 3) the GP appointment, and 4) decision-making around prescribing. Symptomatic relief was a key concern: the most troublesome symptoms were cough, breathing difficulty, fever and malaise. Many parents were reluctant to use self-care medication, tended to support antibiotic use, and believed they are effective for symptoms, illness duration and for preventing complications. However, parental expectations varied from a desire for reassurance and advice to an explicit preference for an antibiotic prescription. These preferences were shaped by: 1) the age of the child, with younger children perceived as more vulnerable because of their greater difficulty in communicating, and concerns about rapid deterioration; 2) the perceived severity of the illness; and 3) disruption to daily routine. When there was disagreement with the GP parents described feeling dismissed, and they were critical of inconsistent prescribing when they re-consult. When agreement between the parent and doctor featured, parents described a feeling of relief and legitimation for consulting, feeling reassured that the illness did indeed warrant a doctor's attention.

Conclusion: Symptomatic relief is a major concern for parents. Careful exploration of expectations, and eliciting worries about key symptoms and impact on daily life will be needed to help parents understand when a no antibiotic recommendation or delayed antibiotic recommendation is made.

For peer review only - http://bmjopen.bmj.com/site/about/guidelines.xhtml

Strengths and limitations of this study

- Provides a detailed view of key experiences and understandings of a sample of parents of children presenting with symptoms of LRTI in southern England.
- Only two fathers included (compared with 23 mothers) so the views of fathers were not fully captured.
- Semi-structured interviews were the optimal data collection method given the aims of the research and analytical saturation was reached.

For peer review only

For peer review only - http://bmjopen.bmj.com/site/about/guidelines.xhtml

BACKGROUND

The majority of respiratory tract infections (RTIs) are viral[1, 2] and will resolve spontaneously with analgesia and rest. Infections and treatment with antibiotics are important health concerns[3]. A third of children presenting to a GP with febrile illness receive an antibiotic prescription[4]. NICE (National Institute for Health and Care Excellence) guidance recommends patients be given no antibiotic prescription or a delayed prescription (to be fulfilled if symptoms worsen)[5], as well as advice about expected time course of infection and how to manage symptoms. Patients' education and general understanding of the medications they are prescribed affect compliance[6] and use of antibiotics leads to greater risk of development of antibiotic resistant bacteria[6, 7]. A US survey study of parents of children under 4 years suggested that parents often believed antibiotics were indicated for cough and upper respiratory tract infections[8], and in yet most parents in the US and UK are aware of antibiotic resistance and its complications[8, 9] or express concern about the overuse of antibiotics. More than half of adults may expect an antibiotic prescription when attending with symptoms of RTI.[6]

Uncomplicated acute lower respiratory tract infection is the commonest acute infection[10], and is likely to have the longest and highest symptom burden of all acute RTIs[11]. Previous work has assessed parents' understanding of the implications and indications for an antibiotic prescription[12] , with parents believing that antibiotics were for more severe illnesses, supported by GPs explanations. This study provides further insight into the tension between GPs and parents in their decision making, and explores key drivers of parental expectation and beliefs about antibiotics.

We report a qualitative study of the key concerns of parents about symptoms of their children presenting with acute LRTI in primary care and their perceptions about antibiotics and the consultation.

METHODS

Participants and procedure

Six research-active practices in the South West of England were identified through the Primary Care Research Network and a poster presentation by CvH at the Wessex Research Sites Initiative Conference. Practices searched their databases for children aged 6 months to 12 years who had presented to primary care with symptoms of LRTI between January 2013

For peer review only - http://bmjopen.bmj.com/site/about/guidelines.xhtml

and March 2015. The recruitment process is shown in Figure 1. A purposive sample was originally intended: however, due to initially slow recruitment, a convenience sampling strategy was used. Whilst this sampling approach can be limiting we recruited a good range of mothers in terms of age and occupation. Both urban and rural practices were invited to participate; however a deprivation scale was used to illustrate variation, as shown in Table 1. Parents were interviewed regardless of the method of GP consultation or whether antibiotics were prescribed.

Figure 1: Recruitment process

Table 1: Practice characteristics and recruitment

Practice	Total letters sent	Positive replies	Negative replies	Total replies	Number consented	Deprivation score*
1	33	1	2	3	1	13.5
2	106	3	1	3	2	19
3	44	3	0	3	2	22.8
4	85	9	3	12	9	7.1
5	12	2	0	2	3	13.6
6	120	7	6	12	6	13.3

******National General Practice Profiles* https://fingertips.phe.org.uk/profile/general-practice/data. **Higher score indicates a higher level of deprivation.**

Interviews

Two female interviewers (CvH and AH) conducted face to face interviews in the participant's home (n=20) and telephone interviews (n=3), each lasting approximately 30 to 60 minutes, with an average duration of 42 minutes. Both interviewers were aware of the NICE guidance regarding antibiotic prescribing for RTIs, and made field notes during interviews. All interviews were audio-recorded and transcribed verbatim. Qualitative interviews provided the best method for gathering insights into parents' views about their children's experiences of RTI and its management in primary care. The interview guide (Appendix 1) included key topic areas, not all of which are covered in this paper. Interviews explored parent views on

For peer review only

whether they would be willing to be randomised in a future study. Its semi-structured design gave flexibility to explore new areas if they arose.

Analysis

Inductive thematic analysis[(13)] was conducted on all transcripts to gain an understanding of the perspectives, attitudes and concerns of parents regarding the managing of LRTI in children in primary care. CvH and AH achieved familiarisation through repeated reading (and listening) of the transcripts. Patterns and themes in the data were identified by CvH and AH and labelled with codes: these code labels referred to the operationalisation of the theme content. A label and full description were provided for each theme. These codes and definitions were refined during a continuous process led by GL, CvH and AH: this involved themes being linked, grouped, moved, re-labelled, added and removed as appropriate in order to produce a set of themes, subthemes, and a coding manual, reviewed and confirmed by the full research team. The coding manual thoroughly explained all the data. .

Participants In total 23 participants took part in this study. In all interviews the mother of the child was interviewed, and fathers were present in two interviews, however anyone who fulfilled a caring role could have participated. The age of the mothers[1] ranged from 21 to 47 (median age 34) and the age of the children ranged from 6 months to 10 years (median age 1 year 9 months) (see Table 2 for participant characteristics).

Table 2: Participant characteristics

Participant	Parent(s) interviewed	Age of mother	Age of child when ill
1	Mother and father	21	6 months
2	Mother	33	3 years
3	Mother	38	10 years
4	Mother	36	1 year
5	Mother	30	1 year
6	Mother	39	1 year
7	Mother	31	2 years

[1] The age of the fathers was not recorded.

For peer review only - http://bmjopen.bmj.com/site/about/guidelines.xhtml

8	Mother	41	6 months
9	Mother	33	1 year
10	Mother	34	1 year
11	Mother	20	2 years
12	Mother	36	8 years
13	Mother	28	9 years
14	Mother and father	39	1 year
15	Mother	47	1 year
16	Mother	33	5-6 months
17	Mother	39	2 years
18	Mother	34	8 years
19	Mother	28	1 year
20	Mother	26	8 years
21	Mother	42	8 years
22	Mother	38	1 year
23	Mother	46	8 years

FINDINGS

Thematic analysis identified four themes relating to factors which enhanced our understanding of parents' perspectives, concerns and experiences of the management of LRTI in children in primary care. Table 3 outlines the themes and subthemes and these are used to structure the findings.

For peer review only

Table 3: Themes identified in analysis

**Themes**	**Sub-themes**
1. Parental perspectives on infection	1.1 Symptoms<br>1.2 Previously similar infections<br>1.3 Parental concerns<br>1.4 Impact of child's illness<br>1.5 Home management strategies and medicine usage
2. Parental perspectives on antibiotic use for lower	2.1 Benefits and efficacy

For peer review only - http://bmjopen.bmj.com/site/about/guidelines.xhtml

respiratory tract infection	2.2 Parental concerns
3. The GP appointment	3.1 Hopes and expectations<br>3.2 Positive/negative experiences<br>3.3 Access to health care<br>3.4 Advice
4. Decision making	4.1 Agreement/disagreement for an AB prescription<br>4.2 Perspectives of GP's prescribing behaviour<br>4.3 Parental knowledge of LRTI and their management

The following sections describe each theme in turn. Numbers in parentheses refer to the theme, subtheme, and relevant exemplary quotation provided in Appendix 2.

1. Parental perspectives on infection

Parental concerns: Parents spoke in detail about their concerns when their child was ill. By far the strongest and most frequent concern centred on breathing difficulties and airway limitations (1.1.1). Other concerns included general concerns or unhappiness that their child was unwell (1.1.2). However, some mothers were less concerned and this was associated with factors such as the child not being their first child, or if the child was perceived to be receiving appropriate treatment (1.1.3). Parents reported worry about symptoms their child had, including the duration of symptoms (1.3.4) or the qualitative difference in the child's behaviour (1.1.5).

Parents of younger children (two years and under) generally had more concerns due to their child's young age. These concerns centred on three areas: increased vulnerability (1.1.6); difficulties in communicating (1.1.7); and the potential for rapid deterioration (1.1.8). Some parents also expressed worry about complications (1.1.9).

Impact of child's illness: Parents were directly asked about the impact of the child's illness on them and their family and readily gave lack of sleep and tiredness across the family as the primary impact (1.2.1). Parents of children with underlying health conditions spoke in more detail about the impact of their child's condition (1.2.2). Working mothers spoke of social implications such as needing additional time off work and the subsequent financial impact

For peer review only - http://bmjopen.bmj.com/site/about/guidelines.xhtml

For peer review only

(1.2.3). These pressures were also reflected when mothers talked about the need for antibiotics to shorten illness duration (see section 2.1).

Symptoms: All symptoms reported by parents are summarised in Table 4, which shows that the most commonly reported symptom by the study participants was a cough, but breathing, fever and malaise were also very commonly reported as troublesome symptoms.

Table 4: Reported symptoms

Symptoms	Frequency
Cough	18
Breathing difficulties	16
Fever/temperature	16
Malaise*	15
Difficulty sleeping	11
Feeding problems/appetite	10
Wheeze	10
Runny nose	9
Fatigue/tiredness	6
Vomit	6
Sore throat/hoarse voice	5
Ear ache	2
Pain	2
Headache	1
Fainting	1
Heart racing	1
Rash	1

*Malaise was used as a term to categorise anything parents said referring to a change in behaviours, character, or appearing unwell (1.1.1).

Previously similar infections: During the course of the interview parents recounted previous infections or the prolonged nature of the infection in question (1.2.1). Mothers of children with underlying health conditions often spoke of this in detail, but it is not central to the current study and so is not reported.

For peer review only - http://bmjopen.bmj.com/site/about/guidelines.xhtml

Home management strategies and medicine usage: Parents initially dealt with their child's infection in different ways. Parents reported trying home management strategies (see Table 5) or complementary medicines before visiting the GP (1.5.1). Parents' views on over-the-counter medicines were varied (1.5.2) although many parents used Calpol and Nurofen[2] to relieve symptoms, most commonly a temperature. Perhaps unsurprisingly many parents reported reluctance to use medications unless they felt it was strongly needed or recommended by a doctor (1.5.3).

Table 5: Home management strategies tried in addition to Calpol / Nurofen:

Strategy	Interview mentioned in
Steam inhalation	4,5,6,8,10,11,13,16
Vaporisers/scents	6,8,13,16
Throat pastilles/cough syrup	3,4,12,14,16,17
Drinks	3,4,8,12,19,21
Cold strategies	13,14,16
Raise the bed	8,16,17,19,23
Vicks	5,8,10,16, 21
Improved diet	5
Other*	2
No additional home management	1,7,9,15,20,22

*Participant 2's child took daily prophylactic antibiotics for a severe undiagnosed health condition: their home management strategy was to give a double dose, as recommended by the specialist.

2. Parental perspectives on antibiotic use for lower respiratory tract infection

Parents spoke in detail about their perceptions of antibiotic use, their attitudes to prescribing and their concerns about antibiotics. Their views were varied but overall parents were happy with their experiences of antibiotic usage in their children.

Benefits and efficacy: Parents cited antibiotics as having three main benefits. Firstly, shortening illness duration (2.1.1), which had the additional benefit of helping parents to return to work (2.1.2). Secondly, antibiotics were described as being able to prevent complications (2.1.3). Thirdly, antibiotics were thought to provide symptomatic relief (2.1.4-2.1.5). Most parents expressed certainty that antibiotics were effective (2.1.6) and they often

[2] Analgesics widely available in the United Kingdom: Calpol contains paracetamol and Nurofen contains ibuprofen and codeine phosphate.

For peer review only - http://bmjopen.bmj.com/site/about/guidelines.xhtml

used their experience of their child recovering during past episodes to infer the effectiveness of antibiotics (2.1.7), although one parent did appreciate there were conditions in which antibiotics are more effective (2.1.8). In contrast, a minority of parents were aware that antibiotics are not always effective while some volunteered awareness of the lack of evidence for prescribing in children (2.1.9).

Parental concerns: Parental concerns about antibiotic use were predominantly about the composition of antibiotic medication (taste, texture, smell, and use of additives). Many parents commented negatively on the taste and texture whereas views on additives such as colourings were more varied. Generally parents had few concerns about adverse effects (2.2.1), especially when their child had not experienced any following previous use (2.2.2).

3. The GP appointment

Hopes and expectations: Parent interviews indicated varied hopes and expectations of the outcome of their GP appointments. Many parents described a desire for symptomatic relief, reassurance and / or advice (3.1.1, 3.1.2) while some, importantly in the minority, attended the GP consultation with an explicit wish for an antibiotic prescription (3.1.3).

Positive and negative experiences:

Many parents had positive views of the GP appointment; in particular when GPs were perceived to take time for a thorough assessment of the child (3.2.1) or with GPs who have built a history with the family (3.2.2). A physical examination has previously been shown to be a key expectation of parents[9]. However, some parents used the interviews as an opportunity to voice frustrations over 'bad' appointments; these centred around feeling rushed, appointments running late or parental concerns not being taken seriously. Differences in parental and GP opinions also contributed to dissatisfaction (discussed further under theme 4).

Access to health care: Parents reported irritation about difficulties in getting appointments. They also discussed a lack of continuity of care, which could mean that deterioration was not noticed by the GP and parents had to recount their story on multiple occasions. Many spoke of needing to use out of hours services, and this brought with it new concerns around queueing, and seeing an unfamiliar doctor in an unfamiliar environment. One parent, living rurally, spoke of concerns around rapid deterioration out of normal working hours. However,

For peer review only - http://bmjopen.bmj.com/site/about/guidelines.xhtml

many parents spoke positively of the use of the NHS 111 service and their advice. Parents of children with underlying health conditions spoke of easy access to medical expertise (3.3.1).

Advice: Parents were asked to discuss advice they were given at the index appointment. They primarily volunteered advice on home-management approaches they had already tried prior to the GP consultation (3.4.1). Many parents did not recall being given specific advice about the natural history of the illness. Of those interviewed some were asked to re-consult if there was no improvement and some recalled receiving advice on how long symptoms would last (3.4.2).

4. Decision making

A shared decision making process between parent and GP, or an explanation of why antibiotics were not necessary, usually led to a more acceptable consultation when parents did not explicitly express a desire for antibiotics.

GP-parent agreement and disagreement about the need for an antibiotic prescription: When there was agreement about the need for a prescription between GP and parents, many parents described a feeling of relief. A prescription seemed to give parents the feeling of illness legitimation[1](4.1.1, 4.1.2). In addition to this, parents reported feeling empowered by a prescription because it signalled a positive action taken to help their child (4.1.3). Parents also described that they were pleased because their child would now have symptomatic relief (4.1.4, 4.1.5). However, parents of children with frequent chest infections or underlying health conditions did not seem to have strong opinions, viewing an antibiotic prescription as a necessary requirement (4.1.6).

When parents felt there was disagreement between them and their doctor, feelings of frustration were described. Frustration that doctors did not agree with their perception of the severity of infection (4.1.7), as there is a perceived link between the need for antibiotics only in severe infections[12],or frustration that doctors did not think antibiotics were indicated (4.1.8). Parents felt frustrated and upset at the lack of symptomatic relief or that they would need to re-consult (4.1.9, 4.1.10).

Parents spoke about feeling dismissed by their GP, especially if the infection was thought to be viral (4.1.11), as seen in previous studies[12]. Parents reported uncertainty when they

For peer review only - http://bmjopen.bmj.com/site/about/guidelines.xhtml

disagreed with the doctor's decision, but this narrative was balanced by belief in ultimately trusting the doctor's judgement and their professional expertise (4.1.12-4.1.14).

Some parents described discomfort and the feeling they could not question the GP's judgement (4.1.15). The majority of parents trusted doctors' expertise but many parents recounted occasions when they had not been prescribed antibiotics initially and then had re-consulted shortly after and been given a prescription. By having to re-consult, parents felt doctors were not consistent, and that their persistence or pestering would result in a prescription. This often seemed to be closely related to dissatisfaction with the GP appointment (4.1.16). Interestingly, it is noted that GPs can feel increased pressure and are more likely to prescribe if a parent has re-consulted[14].

Perspective of GP's prescribing behaviour: Many parents had an awareness of overprescribing antibiotics and its consequences and some commented on doctors clearly trying to limit prescribing (4.2.1). Many parents understood antibiotic resistance to be an idea that over-use would result in your body becoming immune to the antibiotic; an idea which is echoed in the existing literature (4.2.2)[12, 15].

Parental concerns about over-use were reflected in their understanding of antibiotic resistance and superbugs. For example, parents with more accurate scientific knowledge often had a clear appreciation of the consequences of resistance (4.2.3). However, some parents were quite clear that they had very limited understanding of the concept of antibiotic resistance (4.2.4).

Parents offered both positive and negative opinions on GP's prescribing behaviours (4.2.5). Negative opinions of prescribing behaviour were often from word of mouth. For example, a few parents spoke about factors they believed to influence prescribing (4.2.6) such as believing doctors earn more if they prescribe fewer antibiotics (4.2.7).

Parental understanding of LRTI and their management: Parents had a degree of prior knowledge regarding a suggested diagnosis, when to present, and if they felt antibiotics were indicated. Many parents spoke of knowing their child best (4.3.1), although this contrasted with previous comments, where parents described difficulties in communicating with young children. Parents appeared to have had different 'tipping points' with regard to help-seeking,

For peer review only - http://bmjopen.bmj.com/site/about/guidelines.xhtml

For peer review only

such as when to present. Some parents tended to have a particular amount of time they were happy waiting before they consulted to help ensure they received antibiotics (i.e. illness duration would be such that a GP would be more likely to think antibiotics are required) (4.3.2). Others waited a specific number of days (4.3.3) or used a lack of improvement as a reason to consult (4.3.4). Although many parents had clear triggers for consulting, they simultaneously reported concerns about wasting doctors' time (4.3.5), or appearing over-anxious (4.3.6).

Parents often described having a good idea of the diagnosis before presenting. Parental knowledge of a diagnosis came from a variety of experiences: knowing their child, and prior experience, was a chief way of gauging severity and the need to consult (4.3.7). Other parents had conducted internet research to find a diagnosis (4.3.8). Two parents were health care professionals, and relied on their professional expertise (4.3.9) whereas some parents described not having an idea of the likely diagnosis (4.3.10).

There was some confusion over the role of antibiotics in treating LRTI. Some parents understood that antibiotics were only indicated for bacterial infections (4.3.11), whilst others were unclear of the difference between bacterial and viral infections and did not know when antibiotics were indicated (4.3.12), as seen in the current literature[(12)].

DISCUSSION

The study identified four themes that related to parents' perspectives, attitudes and concerns regarding the management of LTRI in children in primary care.

Main findings

Overall, this study provides insight into the views of parents of children who have consulted with their GP for suspected LRTI. It presents up to date evidence of the most significant concerns for parents, primarily sudden deterioration, breathing difficulties or the child's young age. Whilst most believed that antibiotics were effective for the symptoms of LRTI, many parents did not present with a certain expectation for antibiotics, and yet many felt frustration if the GP did not agree with their perception of the need to actively treat their child. There was a tension between parents wanting symptomatic relief for their individual child, the doctor's expertise and their inclination to prescribe based on their understanding of need in the population in general.

For peer review only - http://bmjopen.bmj.com/site/about/guidelines.xhtml

When asked, many parents did not recall being given specific advice on the infection duration. However, interviews revealed that parents did have some understanding about likely duration of a LRTI as shown through the 'tipping points' discussed previously. NICE guidance[5] advises patients should be advised about the natural history of RTIs including usual total length. Interviews suggest this is important for GPs to emphasise so parents can be reassured about when to present.

Comparison with existing literature

Clinicians prescribe for medical indication and other reasons such as perceived pressure from parents[1, 14] but this pressure is not always evident. Inappropriate antibiotic prescriptions may result in problems such as antimicrobial resistance[3, 12] as well as the medicalisation of a self-limiting illness[14, 16]. Whilst this study showed many parents wanted antibiotics, some indicated that they were happy to withhold antibiotic treatment and continue with non-medical self-management strategies and over the counter medicines in a context of reassurance, and when given a sense of illness legitimation. Parental satisfaction was likely to be highest when the GP was perceived to be thorough, that they were listened to and had their concerns taken seriously. Where appropriate, having the medical history of the child or family taken into account also helped with parental satisfaction[14].

This study shows that parents often waited and consulted when they had reached a 'tipping point' at which they felt the severity and duration of the child's illness was such that some action in the form of prescribing antibiotics was warranted. This resonates more generally with research into urinary tract infections[17] in which respondents described an initial reluctance to consult. However, a visit was eventually prompted by symptom severity, often in conjunction with duration. This idea is echoed in a research assessing clinicians' prescribing practice[14]: in the uncertainty of whether this child needs antibiotics, clinicians have their own tipping point. This could be influenced by factors such as whether the parent has previously consulted, proximity to the weekend, or concerns that the parent would not recognise a clinical deterioration. Corroborating evidence with this study lends credibility to our research, adding to this important area in which we need to build a rich understanding. Previous research has identified a need to share knowledge with patients to ensure clear understanding of antibiotics and their appropriate use[12, 15]. However, this present study also showed that even if parents had a good understanding of LRTI and its causes, the perceived

For peer review only - http://bmjopen.bmj.com/site/about/guidelines.xhtml

vulnerability of their child could influence them to present sooner than they may have done had the child been older and more able to communicate how they were feeling.

IMPLICATIONS FOR CLINICAL PRACTICE

Overall, most parents were satisfied with their child's GP consultation. Higher satisfaction was evident in parent narratives when parents believed their concerns about their child's health had been taken seriously, from a thorough examination to having more time spent with them. However, the tension between the parent and GP remains evident[(12)]. Increased GP sensitivity to the wider concerns of parents and the need to employ communication strategies that encourage parental participation and validation of their concerns could ease the path towards improved parent/patient and GP antibiotic discussions and ultimately towards more prudent antibiotic prescribing and use.

The interviews showed that whilst some parents did present to their GP with the expectation of receiving an antibiotic prescription, many parents did not: other aims for the consultation included illness legitimisation and advice for symptomatic relief using home management strategies. GPs need to be aware that parents are often willing to accept a treatment other than antibiotics, and often would rather have an alternative non-antibiotic approach. Providing structured information and exploration of concerns can help negotiate lower antibiotic use[(18))] as it does among adults[(19)]. Parental willingness to consider alternative management strategies often linked with the knowledge parents had about the causes of LRTI and appropriate treatment. Across the interviews, parents displayed varying levels of understanding and beliefs about causes, duration and treatment of LRTI and when they should present. Clarifying patient information, and helping to enhance general understanding, could help to reduce unnecessary GP consultations and smooth the interactional path when parents do consult.

STRENGTHS AND LIMITATIONS

The sample of participants is not representative of the wider population and just sensitises us to key experiences and understandings of a sample of parents. Participants may have had stronger opinions or other reasons for being interviewed than 'typical' or non-participant parents. Only two fathers were included in interviews (compared with 23 mothers) so fathers' views were not fully captured in this study. Interviews occurred at varied times in

For peer review only - http://bmjopen.bmj.com/site/about/guidelines.xhtml

relation to the infection/index consultation for the consulting 'child', which means that some parents had differing lengths of recall, although for most it was only a matter of weeks. Finally, interviews provide insight into perspectives on events not a direct window to the event themselves (such as the GP appointment). However, semi-structured interviews were the optimal data collection method given the aims of this research project.

CONCLUSION

This study aimed to explore factors important to parents in the management of LTRI in children in primary care. Parental views were varied, but clearly indicated that symptomatic relief is a key consideration for their decision-making regarding when to consult and management preferences. Parents do not necessarily expect an antibiotic prescription and a satisfactory consultation can be achieved by being perceived as thorough and in legitimating parents' reason for consulting. There is an on-going need for GPs to explore concerns and expectations carefully, and to tailor advice, information and reassurance for parents - particularly addressing the natural history, worrying symptoms, and the likely impact of antibiotics on symptom severity, duration and complications.

Acknowledgments

Interviewees for their invaluable contributions on this important topic.

NIHR Fellowship funding for Geraldine M Leydon during the conduct of this research.

Ethical approval

Ethical approval for this study was granted by Wales REC 6 reference 14/WA/1226.

Funding

This project was funded by the National Institute for Health Research Health Technology Assessment Programme (project number 13/34/64).

Department of Heath disclaimer:

For peer review only

The views and opinions expressed therein are those of the authors and do not necessarily reflect those of the Health Technology Assessment Programme, NIHR, NHS or the Department of Health.

No further data are available.

There are no competing interests.

Contributors:

GML was responsible for all aspects of the study including design, data analysis and interpretation, and manuscript preparation. AH and CvH collected, analysed and interpreted the data, and prepared the manuscript led by GL. PL is Principal Investigator of a larger affiliated study Antibiotics for lower Respiratory Tract Infection in Children presenting in Primary Care (ARTIC PC) and PL and TV were involved in the generation of the study idea and design, and manuscript comments and revisions.

References:

1. Peters S, Rowbotham S, Chisholm A, Wearden A, Moschogianis S, Cordingley L, et al. Managing self-limiting respiratory tract infections: a qualitative study of the usefulness of the delayed prescribing strategy. Br J Gen Pract. 2011;61(590):e579-89.
2. Thompson PL, Spyridis N, Sharland M, Gilbert RE, Saxena S, Long PF, et al. Changes in clinical indications for community antibiotic prescribing for children in the UK from 1996 to 2006: will the new NICE prescribing guidance on upper respiratory tract infections just be ignored? Arch Dis Child. 2009;94(5):337-40.
3. Davies S, Fowler T, Watson J, Livermore D, Walker D. Annual Report of the Chief Medical Officer: infection and the rise of antimicrobial resistance. Editorial Material. London: Department of Health; 2011 MAY 11 2013. Report No.: 0140-6736 Contract No.: 9878.
4. Moore M. Antibiotics: time to act. British Journal of General Practice. 2013;63(612):340-1.
5. National Institute for Health and Clinical Excellence: Guidance. Respiratory Tract Infections - Antibiotic Prescribing: Prescribing of Antibiotics for Self-Limiting Respiratory Tract Infections in Adults and Children in Primary Care. London: National Institute for Health and Clinical Excellence (UK); 2008.
6. McNulty CA, Nichols T, French DP, Joshi P, Butler CC. Expectations for consultations and antibiotics for respiratory tract infection in primary care: the RTI clinical iceberg. Br J Gen Pract. 2013;63(612):e429-36.
7. Little P, Stuart B, Moore M, Coenen S, Butler CC, Godycki-Cwirko M, et al. Amoxicillin for acute lower-respiratory-tract infection in primary care when pneumonia is not suspected: a 12-country, randomised, placebo-controlled trial. Lancet Infect Dis. 2013;13(2):123-9.
8. Trepka MJ, Belongia EA, Chyou PH, Davis JP, Schwartz B. The effect of a community intervention trial on parental knowledge and awareness of antibiotic resistance and appropriate antibiotic use in children. Pediatrics. 2001;107(1):E6.

For peer review only - http://bmjopen.bmj.com/site/about/guidelines.xhtml

9. Francis N, Phillips R, Wood F, Hood K, Simpson S, Butler C. Parents' and clinicians' views of an interactive booklet about respiratory tract infections in children: a qualitative process evaluation of the EQUIP randomised controlled trial. Bmc Family Practice. 2013;14.

10. Gulliford MC, Dregan A, Moore MV, Ashworth M, Staa T, McCann G, et al. Continued high rates of antibiotic prescribing to adults with respiratory tract infection: survey of 568 UK general practices. BMJ Open. 2014;4(10):e006245.

11. Thompson M, Vodicka TA, Blair PS, Buckley DI, Heneghan C, Hay AD, et al. Duration of symptoms of respiratory tract infections in children: systematic review. BMJ. 2013;347:f7027.

12. Cabral C, Ingram J, Lucas PJ, Redmond NM, Kai J, Hay AD, et al. Influence of Clinical Communication on Parents' Antibiotic Expectations for Children With Respiratory Tract Infections. Annals of family medicine. 2016;14(2):141-7.

13. Braun V, Clarke V. Using thematic analysis in psychology. Qualitative Research in Psychology [Internet]. 2006 05.01.15; 3(2):[77-101 pp.].

14. Horwood J, Cabral C, Hay AD, Ingram J. Primary care clinician antibiotic prescribing decisions in consultations for children with RTIs: a qualitative interview study. The British journal of general practice : the journal of the Royal College of General Practitioners. 2016;66(644):e207-13.

15. Brookes-Howell L, Elwyn G, Hood K, Wood F, Cooper L, Goossens H, et al. 'The Body Gets Used to Them': Patients' Interpretations of Antibiotic Resistance and the Implications for Containment Strategies. Journal of General Internal Medicine. 2012;27(7):766-72.

16. Andrews T, Thompson M, Buckley DI, Heneghan C, Deyo R, Redmond N, et al. Interventions to Influence Consulting and Antibiotic Use for Acute Respiratory Tract Infections in Children: A Systematic Review and Meta-Analysis. Plos One. 2012;7(1).

17. Leydon GM, Turner S, Smith H, Little P, team U. Women's views about management and cause of urinary tract infection: qualitative interview study. BMJ. 2010;340:c279.

18. Francis NA, Butler CC, Hood K, Simpson S, Wood F, Nuttall J. Effect of using an interactive booklet about childhood respiratory tract infections in primary care consultations on reconsulting and antibiotic prescribing: a cluster randomised controlled trial. British Medical Journal. 2009;339.

19. Little P, Stuart B, Francis N, Douglas E, Tonkin-Crine S, Anthierens S, et al. Effects of internet-based training on antibiotic prescribing rates for acute respiratory-tract infections: a multinational, cluster, randomised, factorial, controlled trial. Lancet. 2013;382(9899):1175-82.

For peer review only

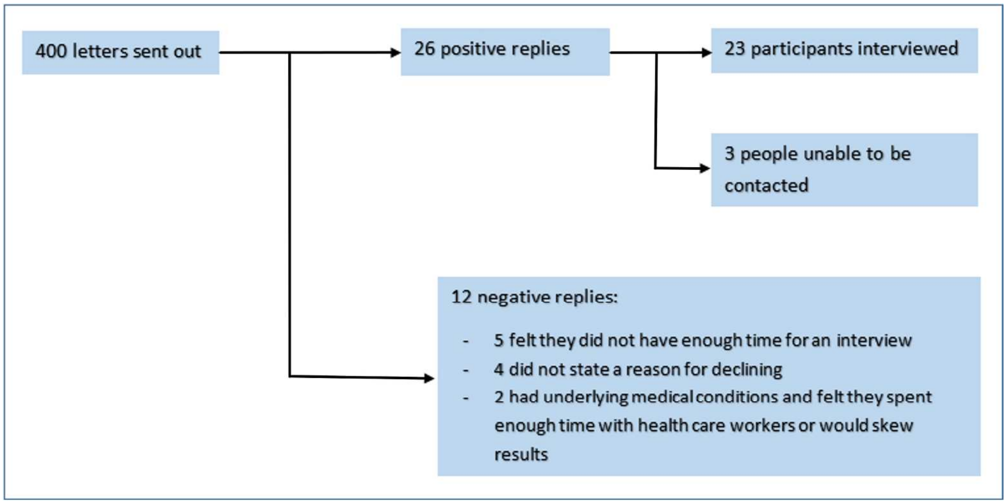

Recruitment process

203x100mm (300 x 300 DPI)

For peer review only

Appendix 1: Interview guide

Section 1: presenting illness

- Before visiting the GP can you describe what happened/when you first noticed [name] was poorly?
- By the time you took [name] to the GP what particular symptoms did [name] have?
  - What, timings, together/separate?
- How did you feel about [name] being ill at the time?
- What was it that made you take [name] to the GP?
  - What was most concerning about the illness?
    - Eg compared to other coughs/illness
  - Had they had any previously similar episodes?
    - Generally quite well?
- Can you describe what you thought might be wrong with [name] at the time?
  - Read anything on the internet
  - Advice from friends/family/pharmacist
- How were you feeling in yourself?
  - Did [name's] illness impact on the family?
  - Concerns re cross-infection?
- Before visiting the GP had you tried any form of treatment?
  - E.g Calpol, Nurofen?
  - Talk to other parents?
  - Generally do you wait and see/get medicines?
- What happened at your appointment?
  - What did the GP do?
  - Did they give you any medicines/advice (and if so what) /examine [name]?
- What did you hope to get from the GP appointment?
  - Reassure, prescribe antibiotics
- Can you think back and describe whether your hopes/expectations about the GP consultation were met?
  - Why / why not?
- If [name] had the same symptoms again how would you manage the situation next time?
  - Would you do anything differently?
    - Visit GP sooner?
    - Delay?

For peer review only - http://bmjopen.bmj.com/site/about/guidelines.xhtml

        - Not attend?
        - Make wishes more explicit with GP?
        - Reconsider value of Abs?

Section 2: understanding of antibiotics

- [Name] was/wasn't prescribed a/bs, how did you feel about that?
    - Why did you think a/bs were/were not needed?
    - E.g. Did you want [name] to have antibiotics?
    - How did antibiotics help [name] and your family get better?
        - Quicker recovery
        - Feel empowered
        - Could go back to school/playgroup?
    - How would you have felt if [name] had not received antibiotics?
        - Can you think of a time you have taken [name] to the GP and they weren't given a/bs?
        - Do you think the a/bs made [name] better?
        - Would they have got better themselves with no a/bs?
- Can you tell me about your thoughts about antibiotics and their use in children with chest infections?
    - E.g. effective/not-effective? Best avoided?
    - E.g. any concerns about giving antibiotics to children?
    - Side effects
    - Read anything of the Internet etc?
    - Concerns about sugar content/flavourings/colouring?
    - Taste issues?
- Have you heard of antibiotic resistance?
    - What does it mean to you?
    - Check understanding re resistant bacteria vs resistant body
- Have you heard about 'super-bugs'? Do you see this to be linked to prescribing antibiotics?
- Was it explained to you that you must finish all the antibiotics prescribed? And why it is important?

- [Remind parents that it does not matter if they are unsure, and provide information leaflet explaining antibiotic resistance after the interview.]

For peer review only - http://bmjopen.bmj.com/site/about/guidelines.xhtml

Recruitment/participation:

- A future trial is currently being developed to test how effective antibiotics are because we need to know which antibiotics work best in which children. We know that whilst most children get antibiotics, many children will probably settle without antibiotics but some will benefit from antibiotics. What we don't know is if antibiotics help or not and which children get benefit from antibiotics and which children suffer side effects (rashes, diarrhoea, resistance).
- The team are going to be doing a trial which would involve some children taking the medicine and others taking a dummy/sugar-coated pill, this would be randomly allocated. All the children will be fully monitored during the trial and will be carefully reviewed if they are not improving.
    - If you had the opportunity would you be interested in participating in this kind of trial?
    - What do you think about the possibility of your child taking the dummy pill?
- How did you feel about being contacted by letter?
    - Preferred GP/face to face?

For peer review only - http://bmjopen.bmj.com/site/about/guidelines.xhtml

Appendix 2: Exemplary Quotations

Themes	Illustrative quotations from participants
1. Parental perspectives on infection	
1.1 Parental concerns	1.1.1 "Oh, terrified. Because of the breathing, I was really frightened about – because the coughing – when she's having these coughing fits – it's just really scary and I was – I was scared to put her to bed because of the wheezing" (P4, 106-108)<br>1.1.2 "all you want to do is just stop the cough and you can't" (P21, 83)<br>1.1.3 "Fine, obviously just a little bit concerned, that was all; but apart from that, I was fine – as long as she was getting the treatment that she needed, then I was happy" (P1, 71-72)<br>1.1.4 "she'd been unwell really for the biggest part of the beginning of the week" (P22, 19)<br>1.1.5 "I think my main concern was the fact that he wasn't his usual – playful self" (P9, 34)<br>1.1.6 "where it's – such a small body and it's so hard for them to actually fight back" (P10, 142-3)<br>1.1.7 "It's – the problem we have with [name] is it's very hard to know when he is unwell because he just carries on and when – where he's quite young as well – he won't sit there and say, mummy my throat hurts, he'll just carry on as normal" (P7, 30-32)<br>1.1.8. "You know, because he's only little as well and at that age – because they can go downhill very quickly, that's what I was worried about" (P8, 89-90).<br>1.1.9 "there was that fear that it could have – it could be developing something worse" (P13, 302-303)
1.2 Impact of child's illness	1.2.1 "[son] did hear her coughing, so it did keep him awake as well" (P21, 313-314)<br>1.2.2 "she gets very out of breath very quickly, so she can't join in her sports days, things like that; she doesn't ever complete a full week at pre-school" (P2, 109-110)<br>1.2.3 "a lot of times parents are working, both parents, you need to get back to work; it costs money to have a child off sick and then you have to stay off sick, you lose a day's pay or – or perhaps you have to pay a childminder, even though you're not at work, you have to stay home from work as well" (P10, 286-289)
1.3 Symptoms	1.3.1 "He wasn't his usual playful self, he was just sort of a bit off colour" (P9, 21-22)
1.4 Previously similar infections	1.4.1 "Well she's had – antibiotics now for chest infections probably three or four times" (P14, 10)
2. Parental perspectives on antibiotic use for lower respiratory tract infection	
2.1 Benefits and efficacy	2.1.1 "If I didn't give her the antibiotics, I think maybe she would of, but it probably would have tooken a lot, lot longer, but…no, I think the antibiotics helped. They helped – and they make it quicker as well, so they are not so ill for long" (P1, 173-175)<br>2.1.2 "anything you can do to help speed up the process and get her better, so that you can get back to normal, because it's – it has such an impact" (P4, 327-329)<br>2.1.3 "I think the risks of something bad happening or, you know, in the night and not being able to breathe – would…yes, would have been more worrying and, you know maybe either I would have had to have called an ambulance or taken him into hospital or something like that. And I think that was definitely avoided by the antibiotics" (P5, 284-289)<br>2.1.4 "If there's antibiotics that can fix whatever they've got then – yes – that they can sleep better and breath" (P5, 193-4)<br>2.1.5 "I don't think there's any point in letting a little baby cough like that and suffer like that for – I don't know how long – I – worry that they won't – not that they won't get over eventually themselves, but – I think..that it's my job to keep them well and – give them – yes – the opportunity to get better" (P5, 202-5)

For peer review only - http://bmjopen.bmj.com/site/about/guidelines.xhtml

	2.1.6 "They 100% work, 100%" (P1, 231)<br>2.1.7 "I mean they made [name] better in December, so, yes, they must be effective in children" (P3, 280-281)<br>2.1.8 "I do, yes. Antibiotics certainly helped both my children – but I think they are only effective if you use them correctly, so if you do finish the whole course of treatment" (P10, 275-276)<br>2.1.9 "should he be having antibiotics, you know – is it going to make any difference at all?" (P9, 85-86)
2.2 Parental concerns about problems with antibiotics	2.2.1 "no, upset like nasty nappies and things but – no – nothing, aside from that" (P18, 314-315).<br>2.2.2 "no, none that I'm aware of" (P23, 161)
3. *The GP appointment*	
3.1 Hopes and expectations	3.1.1 "Just to get her better really" (P21, 179)<br>3.1.2 "just that I wasn't a neurotic mother, confirmation that there was something wrong with my child and something to fix her" (P17, 125-6).<br>3.1.3 "I was hoping for antibiotics. I was – I kind of knew that it was going to be antibiotics, again, just going from experience" (P7, 180-1).
3.2 Positive / negative experiences	3.2.1 "the lady we saw the next day was lovely; she really took the time to…you know, really – sort of – looked at everything and – seemed, you know, genuine, genuinely – sort of concerned for her and what have you" (P4, 214-217)<br>3.2.2 "I suffer with postnatal depression, so that's why I like – she [GP] understands me as a mother and a person as well and there are a couple of other doctors that I like to see because they know the history" (P18, 173-176).
3.3 Access to health care	3.3.1 "It's nice to know that I can – if I do have any questions, I can phone up and speak to the nurses and they've always said, you know, no question is too silly, we are always at the end of the phone" (P7, 305-7)
3.4 Advice	3.4.1 "Spoke to me through what to do, the best thing to do, you know, keeping him cool, giving me plenty of information as well" (P19, 190-191)<br>3.4.2 "I think she probably said that we'd see some improvement within about four or five days" (P14, 222-3)
4. *Decision making*	
4.1Agreement/disagreement for an antibiotic prescription	4.1.1 "I must admit there was a little bit of relief – because it meant I hadn't overreacted and I think that she was poorly enough" (P3, 205-6).<br>4.1.2 "I think – I felt like she'd taken his illness seriously" (P10, 191).<br>4.1.3 "It felt like – good, you know something – you feel like something is being done to help the situation" (P4, 300-301).<br>4.1.4 "I know that he'd get better with them" (P20, 149)<br>4.1.5 "I was quite happy for her to have antibiotics to treat it because it was disturbing her sleep and showing no signs of dissipating by itself (P17, 35-36)<br>4.1.6 "…it was kind of expected really, neither happy nor unhappy" (P15, 153)<br>4.1.7 "But you don't see them every day, every night, how the kid's suffering or what they were doing, you know" (P12, 414-416)<br>4.1.8 "I was a bit shocked I thought, oh, right, okay, it's a cough – because obviously there's something not right, he's got a cough" (P8, 229-230)<br>4.1.9 "I think it was upsetting because I was thinking that my child would be suffering" (P10, 218-9).<br>4.1.10 "I think I would have been a little frustrated because at that point I did know that it was – it was getting worse and I probably would have thought – well it's going to need another trip to the doctor's in a couple of days – which is just a waste of time for me and I probably would have just been quite annoyed" (P9, 239-242)<br>4.1.11 "they'd sent me away saying she just had a virus" (P4, 68-9)<br>4.1.12 "…I wasn't convinced that they were right…really, because…if he's going to clear it up on his own, it's normally gone within three or four days […] But – yes, doctors know what they're talking about, don't they? You just go with it, don't you – really" (P16, 234)<br>4.1.13 "So, like I said, I go by the doctor's judgement to make it better for my daughter" (P1, 148-9)

For peer review only - http://bmjopen.bmj.com/site/about/guidelines.xhtml

	4.1.14 "I'd probably let them tell me what's best for her, really. They are the professionals, I'm not" (P21, 216-217)<br>4.1.15 "in some cases you don't feel like you can challenge a doctor because they think well, they are the doctor, they're trained and if I start kicking off and making a fuss, they're just going to get really annoyed with me and just think I'm an overprotective parent" (P7, 278-281).<br>4.1.16 "I was here [GP surgery] yesterday, they told me there is no chest infection: why do I have to wait for another night for him to be treated? If he had been given [antibiotics] yesterday, they could – he could have been better, you know" (P12, 322-325)
4.2 Perspectives of GP's prescribing behaviour	4.2.1 "I'm of the generation that – you know – if you've got an infection, go to the doctor and get some antibiotics. I know now that – that things are very different" (P22, 349-351)<br>4.2.2 "Um…is where your body can't fight – you like become immune to the antibiotics and it doesn't do anything?" (P13, 455-456)<br>4.2.3 "Yes, overprescribing antibiotics or prescribing them for things like coughs and colds – which they are ineffective against anyway…So that can lead to superbugs" (P10, 376-7).<br>4.2.4 "No, never heard of it" (P11, 176)<br>4.2.5 "I think, in my experience, there's a good balance here – about prescribing them when they are needed" (P5, 431-432)<br>4.2.6 "I have heard of people going to the GP for really minor things and getting antibiotics and I find that frustrating" (P10, 233-234).<br>4.2.7 "There was a thing on the news the other day, that they're…doctors are going to earn more money if they prescribe less antibiotics or something. That's wrong, isn't it?" (P16, 110-111).
4.3 Parental knowledge of LRTI and their management	4.3.1 "you know instantly if your child is not right" (P3, 72)<br>4.3.2 "I know that they are going to – tell you to wait a week, so I wait a week before I go in" (P16, 255-6).<br>4.3.3 "three days is too much continuously, then I have to bring them in" (P12, 399-400)<br>4.3.4 "when they are getting worse and no sign of getting better, then I would, without doubt, go" (P5, 242-243)<br>4.3.5 "I feel so guilty to think – oh God – I feel like I'm wasting their time" (P8, 223)<br>4.3.6 "You sometimes feel a little bit like…a neurotic mother" (P4, 211)<br>4.3.7 "Yes, I know the signs: he has them so often that I can sort of pick the signs and as soon as that sign appears" (P11, 38-9)<br>4.3.8 "As a parent, you think the worst; you start Googling. I think – we knew it was some kind of chest infection" (P13, 152-4).<br>4.3.9 "I'm a nurse, so I kind of have quite a good understanding of what was going on" (P9, 71).<br>4.3.10 "I did think she had a cold and obviously until the said what it is, then I was obviously a bit shocked" (P1, 68-9)<br>4.3.11 "antibiotics shouldn't just be given out for coughs and colds and viruses and things…I know that GPs often say – antibiotics are no good for viruses, it's only bacterial infections" (P10, 246-7)<br>4.3.12 "because obviously a chest infection is obviously a viral infection, isn't it, so I think most infections to need something to help" (P1, 132-133)

For peer review only - http://bmjopen.bmj.com/site/about/guidelines.xhtml

COREQ (COnsolidated criteria for REporting Qualitative research) Checklist

A checklist of items that should be included in reports of qualitative research. You must report the page number in your manuscript where you consider each of the items listed in this checklist. If you have not included this information, either revise your manuscript accordingly before submitting or note N/A.

Topic	Item No.	Guide Questions/Description	Reported on Page No.
**Domain 1: Research team and reflexivity**			
Personal characteristics			
Interviewer/facilitator	1	Which author/s conducted the interview or focus group?	5
Credentials	2	What were the researcher's credentials? E.g. PhD, MD	Author bios
Occupation	3	What was their occupation at the time of the study?	
Gender	4	Was the researcher male or female?	5
Experience and training	5	What experience or training did the researcher have?	Not reported
Relationship with participants			
Relationship established	6	Was a relationship established prior to study commencement?	NA
Participant knowledge of the interviewer	7	What did the participants know about the researcher? e.g. personal goals, reasons for doing the research	NA
Interviewer characteristics	8	What characteristics were reported about the inter viewer/facilitator? e.g. Bias, assumptions, reasons and interests in the research topic	Not reported
**Domain 2: Study design**			
Theoretical framework			
Methodological orientation and Theory	9	What methodological orientation was stated to underpin the study? e.g. grounded theory, discourse analysis, ethnography, phenomenology, content analysis	
Participant selection			
Sampling	10	How were participants selected? e.g. purposive, convenience, consecutive, snowball	5
Method of approach	11	How were participants approached? e.g. face-to-face, telephone, mail, email	5
Sample size	12	How many participants were in the study?	5
Non-participation	13	How many people refused to participate or dropped out? Reasons?	5
Setting			
Setting of data collection	14	Where was the data collected? e.g. home, clinic, workplace	6
Presence of non-participants	15	Was anyone else present besides the participants and researchers?	5
Description of sample	16	What are the important characteristics of the sample? e.g. demographic data, date	7
Data collection			
Interview guide	17	Were questions, prompts, guides provided by the authors? Was it pilot tested?	20
Repeat interviews	18	Were repeat inter views carried out? If yes, how many?	NA
Audio/visual recording	19	Did the research use audio or visual recording to collect the data?	6
Field notes	20	Were field notes made during and/or after the inter view or focus group?	5
Duration	21	What was the duration of the inter views or focus group?	6
Data saturation	22	Was data saturation discussed?	3
Transcripts returned	23	Were transcripts returned to participants for comment and/or	NA

For peer review only

Topic	Item No.	Guide Questions/Description	Reported on Page No.
		correction?	
**Domain 3: analysis and findings**			
Data analysis			
Number of data coders	24	How many data coders coded the data?	6
Description of the coding tree	25	Did authors provide a description of the coding tree?	No
Derivation of themes	26	Were themes identified in advance or derived from the data?	6
Software	27	What software, if applicable, was used to manage the data?	NA
Participant checking	28	Did participants provide feedback on the findings?	NA
Reporting			
Quotations presented	29	Were participant quotations presented to illustrate the themes/findings? Was each quotation identified? e.g. participant number	23
Data and findings consistent	30	Was there consistency between the data presented and the findings?	8
Clarity of major themes	31	Were major themes clearly presented in the findings?	8
Clarity of minor themes	32	Is there a description of diverse cases or discussion of minor themes?	8

Developed from: Tong A, Sainsbury P, Craig J. Consolidated criteria for reporting qualitative research (COREQ): a 32-item checklist for interviews and focus groups. *International Journal for Quality in Health Care*. 2007. Volume 19, Number 6: pp. 349 – 357

Once you have completed this checklist, please save a copy and upload it as part of your submission. DO NOT include this checklist as part of the main manuscript document. It must be uploaded as a separate file.

For peer review only
